# A Nucleic-Acid Hydrolyzing Single Chain Antibody Confers Resistance to DNA Virus Infection in HeLa Cells and C57BL/6 Mice

**DOI:** 10.1371/journal.ppat.1004208

**Published:** 2014-06-26

**Authors:** Gunsup Lee, Jaelim Yu, Seungchan Cho, Sung-June Byun, Dae Hyun Kim, Taek-Kyun Lee, Myung-Hee Kwon, Sukchan Lee

**Affiliations:** 1 Department of Genetic Engineering, Sungkyunkwan University, Jangan-gu, Suwon, Korea; 2 Fruit Research Division, National Institute of Horticultural and Herbal Science, Rural Development Administration, Suwon, Korea; 3 Animal Biotechnology Division, National Institute of Animal Science, Rural Development Administration, Suwon, Korea; 4 South Sea Environment Research Department, Korea Institute of Ocean Science and Technology, Geoje, Korea; 5 Department of Microbiology, Ajou University School of Medicine, San 5, Woncheon-dong, Yeongtong-gu, Suwon, Korea; University of North Carolina at Chapel Hill, United States of America

## Abstract

Viral protein neutralizing antibodies have been developed but they are limited only to the targeted virus and are often susceptible to antigenic drift. Here, we present an alternative strategy for creating virus-resistant cells and animals by ectopic expression of a nucleic acid hydrolyzing catalytic 3D8 single chain variable fragment (scFv), which has both DNase and RNase activities. HeLa cells (SCH7072) expressing 3D8 scFv acquired significant resistance to DNA viruses. Virus challenging with Herpes simplex virus (HSV) in 3D8 scFv transgenic cells and fluorescence resonance energy transfer (FRET) assay based on direct DNA cleavage analysis revealed that the induced resistance in HeLa cells was acquired by the nucleic acid hydrolyzing catalytic activity of 3D8 scFv. In addition, pseudorabies virus (PRV) infection in WT C57BL/6 mice was lethal, whereas transgenic mice (STG90) that expressed high levels of 3D8 scFv mRNA in liver, muscle, and brain showed a 56% survival rate 5 days after PRV intramuscular infection. The antiviral effects against DNA viruses conferred by 3D8 scFv expression in HeLa cells as well as an *in vivo* mouse system can be attributed to the nuclease activity that inhibits viral genome DNA replication in the nucleus and/or viral mRNA translation in the cytoplasm. Our results demonstrate that the nucleic-acid hydrolyzing activity of 3D8 scFv confers viral resistance to DNA viruses *in vitro* in HeLa cells and in an *in vivo* mouse system.

## Author Summary

Most strategies for developing virus-resistant transgenic cells and animals are based on the concept of virus-derived resistance, in which dysfunctional virus-derived products are expressed to interfere with the pathogenic process of the virus in transgenic cells or animals. However, these viral protein targeting approaches are limited because they only target specific viruses and are susceptible to viral mutations. We describe a novel strategy that targets the viral genome itself, rather than viral gene products, to generate virus-resistant transgenic cells and animals. We functionally expressed 3D8 scFv which has both DNase and RNase activities, in HeLa cells and transgenic mice. We found that the transgenic cells and mice acquired complete resistance to two DNA viruses (HSV and PRV) without accumulating the virus, and showed delayed onset of disease symptoms. The antiviral effects against DNA viruses demonstrated in this study were caused by (1) DNase activity of 3D8 scFv in the nucleus, which inhibited DNA replication or RNA transcription and (2) 3D8 scFv RNase activity in the cytoplasm, which blocked protein translation. This strategy may facilitate control of a broad spectrum of viruses, including viruses uncharacterized at the molecular level, regardless of their genome type or variations in gene products.

## Introduction

Viruses are pathogenic agents that cause potentially devastating diseases such as the flu, hepatitis, poliomyelitis, acquired immunodeficiency syndrome (AIDS), severe acute respiratory syndrome (SARS), avian influenza, and foot-and-mouse disease [1], [2], [3]. Many antiviral drug studies have been based on a functional analysis of viral genes and an understanding of the virus life cycle. McFarland and Hill (1987) showed successful vaccination of mice and pigs with a mutant PRV thymidine kinase [4]. Qing Ge also demonstrated that nucleocapsid siRNA or a component of the RNA transcriptase (PA) is a good antiviral drug to protect against influenza virus by inhibiting viral RNA transcription with siRNAs [5]. In addition, acyclovir, which is the best antiviral agent against HSV-1, is a nucleotide analogue that shows an antiviral effect by inhibiting DNA replication [6]. However, commercially-developed antiviral drugs such as viral DNA polymerases, viral reverse transcriptases, and neuraminidase inhibitors target one or two viruses [7], [8], [9], [10], [11]. Thus, a new strategy is needed to prepare for outbreaks caused by new viruses or new mutant viruses because of the high mutation rates of viral genomes and recombination events among closely-related viruses [12], [13].

A scFv is a recombinant antibody fragment, which commonly consists of a full variable region of an immunoglobulin heavy chain covalently linked to the corresponding variable region of an immunoglobulin light chain. scFvs have multiple benefits over traditional monoclonal antibodies due to their greatly reduced size, ease of genetic manipulation, and production of antibodies against viral proteins [14]. In 1994, scFv which binds to the Human immunodeficiency virus 1 (HIV-1) regulatory protein Rev was expressed intracellularly and potently inhibited HIV-1 replication in scFv immunized cells [15]. In addition, scFv against HIV integrase and reverse transcriptase showed reduced viral progeny in virus infected cells [16], [17]. The retroviral capsid protein can be used as an antiviral target and thereby extend the number of targets that can potentially be used in combined scFv-based gene therapy approaches [18]. However, despite the many virus resistant studies using scFv proteins, no reports are available about scFv having an antiviral effect against a broad spectrum of viruses.

Montandon et al. (1982) showed antiviral effects against Moloney murine leukemia virus (M-MuLV) with DNase I. In another study, DNase I digested DNA in the form of unmethylated proviral M-MuLV selectively [19]. Exonuclease, ISG20, which is induced by type 1 interferon (IFN), was over-expressed in CEL cells to inhibit HIV replication through nuclease activity [20], [21]. Another case was reported in a plant system using Pac1, an RNase isolated from the yeast *Schizosaccharomyces pombe*. Challenging a transgenic potato that expresses Pac1 with potato spindle tuber viroid (PSTVd) resulted in suppression of PSTVd infection and accumulation without significant undesired effects on the plant. The anti-viral effects of these RNases depend on their RNA-hydrolyzing activity [22].

We have shown previously that the 3D8 scFv protein has nuclease activity that non-specifically degrades *in vitro* DNA and RNA substrates [23]. 3D8 scFv originated from MRL mice and is a recombinant single chain antibody linked to VH and VL by linker peptides [14]. 3D8 scFv has catalytic activity supporting the hydrolysis of both single stranded and double stranded DNAs in the presence of Mg^2+^ without significant sequence specificity. In addition, efficient degradation of RNAs by 3D8 scFv in the presence of EDTA demonstrates that 3D8 scFv does not need divalent metal ions for its RNase activity [23]. Additionally, 3D8 scFv has DNase and RNase activity but when the active sites of each VL and VH are changed from histidine (35 position in VH and 94 position in VL) to alanine, respectively, by site-directed mutagenesis, the mutant 3D8 scFv loses DNase activity but retains RNase activity [24]. We previously showed that 3D8 scFv can be applied to protect PK15 cells from virus infection. PK15 cells harboring 3D8 scFv show resistance to *classical swine fever virus* (CSFV) through RNase activity [25]. Also, we previously found that progenies of the transgenic tobacco plant acquired complete resistances against two single stranded (ss)-DNA geminiviruses, four ssRNA tobamoviruses, and one ssRNA cucumovirus [26], [27]. So far, chemicals or specific antibodies for viral proteins have been mostly used in antiviral agents. But, 3D8 scFv with a completely different mechanism than antiviral agents is expected to be effective against a broad spectrum of viral infections.

We established *in vitro* cell and *in vivo* mouse systems harboring 3D8 scFv genes and antiviral mechanism against PRV and HSV as DNA viruses with dsDNA genomes. The expression patterns of viral open reading frames (ORFs) were identified and the FRET assay and confocal microscopy were performed to investigate the antiviral mechanism shown in our experimental *in vitro* and *in vivo* systems. Taken together, our data support that the antiviral effect against the DNA virus used in this work was caused by (1) nuclear DNase activity that inhibited DNA replication or RNA transcription and (2) RNase activity in the cytoplasm blocked protein translation.

## Results and Discussion

### The 3D8 scFv cell line expresses 3D8 proteins in both the cytosol and nucleus

**Figure 1:**
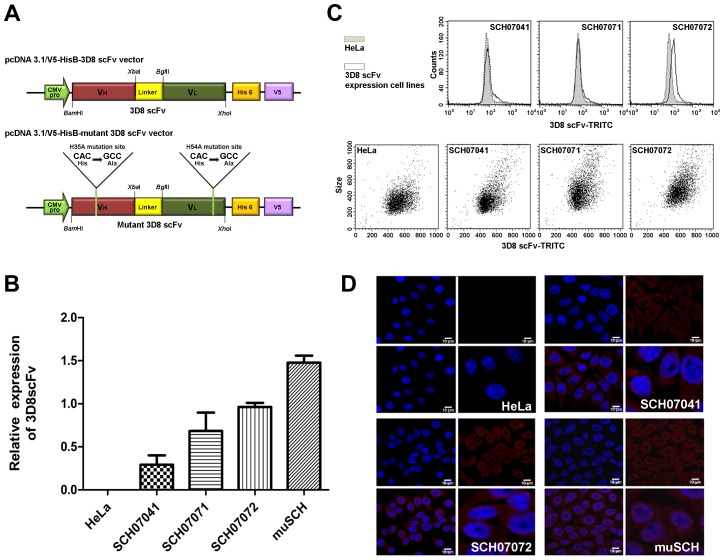
Construction of 3D8 scFv-expressing HeLa cells. **A**. Schematic diagrams of the plasmid constructs. Construction of the (a) pcDNA3.1/V5-HisB-3D8 scFv vector and (b) pcDNA3.1/V5-HisB-mu3D8 scFv vector. Yellow line is the mutation site (His→Ala) **B**. 3D8 scFv expression levels were analyzed by quantitative real-time polymerase chain reaction (PCR) in transgenic cell lines. The relative concentrations of 3D8 scFv were calculated after normalization to the GAPDH gene using the delta delta C_T_ method. Data bars represent mean ± standard error. The expression levels of each cell line were compared to SCH07072. **C**. Identification of three 3D8 scFv cell lines (SCH07041, SCH07071, and SCH07072) by flow cytometry. Transgenic and wild-type HeLa cells were stained with 3D8 scFv Ab and TRITC-anti-rabbit Ig for flow cytometry. **D**. Localization of 3D8 scFv in transgenic and wild-type HeLa cells by immunocytochemistry. Nuclei were detected by DAPI staining (blue). 3D8 scFv expression was monitored by immunofluorescence using a polyclonal anti-3D8 scFv antibody that was visualized with TRITC (Rhodamine Red). 3D8 scFv proteins were localized in both the cytosol and nucleus of SCH07072 and muSCH cells.

The gene encoding 3D8 scFv was introduced into HeLa cells to test whether 3D8 scFv expressing cell lines could protect against DNA virus (HSV-1 and PRV) infection. The DNAs coding for both the wild-type 3D8 scFv protein (SCH) and the inactive DNase-mutated 3D8 scFv protein (muSCH) were cloned under the transcriptional dependence of the cytomegalovirus promoter in the pcDNA3.1/V5-HisB vector (**Figure 1A**). Three 3D8 scFv lines (SCH07041, SCH07071, and SCH07072) and mutated 3D8 scFv (muSCH) were selected by serial dilution in media containing G418 [28]. We evaluated 3D8 scFv expression levels in selected cell lines by quantitative real-time reverse transcription polymerase chain reaction (RT-PCR), flow cytometric analysis, and immunocytochemistry. According to quantitative real-time RT-PCR, SCH07072 expressed the highest 3D8 scFv RNA compared to that of the other two cell lines (1.3 and 4 times more). The mutant 3D8 scFv-transfected (muSCH) cells expressed similar levels of 3D8 scFv RNA to SCH07072 (**Figure 1B**). Flow cytometry data revealed that when the mean for the HeLa cell line as a control was 79.48, SCH07041, SCH07071, and SCH07072 were 88.45, 109.17, and 126.38, respectively (Geo mean: HeLa = 74.51, SCH07041 = 76.88, SCH07071 = 89.89, and SCH07072 = 115.33) and was similar to the results shown in the quantitative real-time RT-PCR analysis (**Figure 1C**). Confocal microscopy demonstrated that 3D8 scFv proteins in SCH07072 and muSCH were targeted and localized in both the cytosol and nucleus (**Figure 1D**). Common features of anti-DNA antibodies that have a large number of positively-charged residues within complementarity determining regions (CDRs) of VH or VL domains due to their antigen binding properties, which resemble the nuclear localization signal (NLS), have been attributed to their final accumulation within the nucleus of cells, such as cell-penetrating peptides (CPPs) [29]. Therefore, 3D8 scFv proteins were localized in the cytosol using a vector system and targeted to the nucleus by a NLS.

### 3D8 scFv exhibits resistance to HSV type1 and PRV in stably transfected cell lines

The three 3D8 scFv lines and the one mutant 3D8 scFv line were challenged with HSV-1::GFP and PRV which had dsDNA as a genome. Virus-infected HeLa cells express GFP in the cytoplasm after HSV infection [30] and PRV infection results in cytopathic effects (CPE) in HeLa cells. In addition, PRV results in multinuclear giant cell formation as a PRV CPE [31].

**Figure 2:**
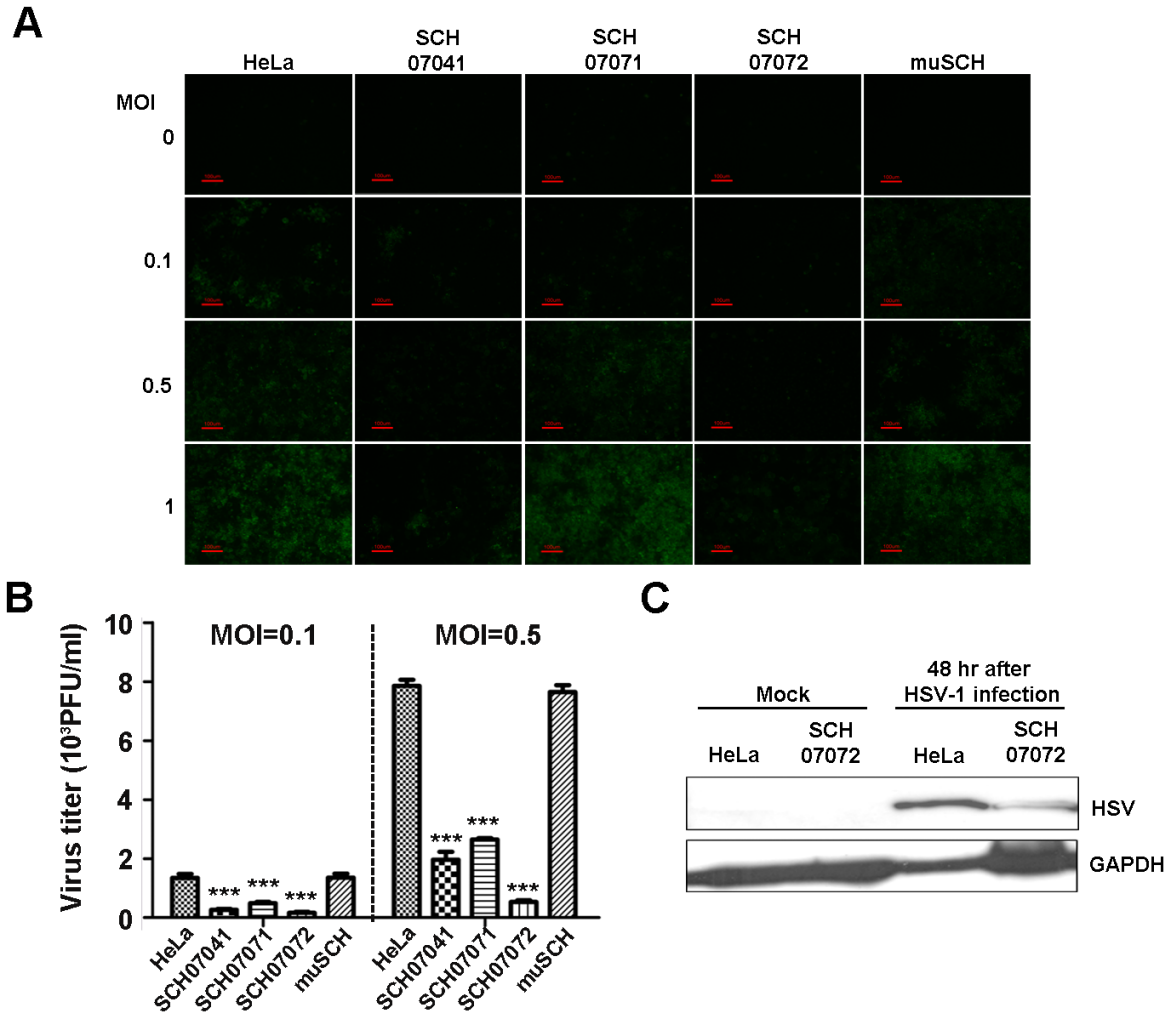
3D8 scFv expression in transgenic HeLa cells confers resistance to HSV infection. **A**. Three 3D8 scFv cell lines and one mutant 3D8scFv cell line were challenged with HSV::GFP at different MOIs (0.1, 0.5, and 1). The SCH07072 line showed the lowest GFP expression compared to that of the other lines (SCH07041, SCH07071, and muSCH). **B**. Wild-type HeLa, SCH07072, and muSCH cells were infected with HSV::GFP at 0.1 and 0.5 MOI. Plaque assays revealed that SCH07072 cells had the lowest amount of virus progeny (0.1 MOI: HeLa = 1,343.33±139.68, SCH07041 = 266.67±31.80, SCH07071 = 483.33±43.72, SCH07072 = 163.33±29.06, muSCH = 1,356.67±131.70; 0.5 MOI: HeLa = 7,851.00±209.10, SCH07041 = 1,964.00±263.04, SCH07071 = 2,649.67±49.02, SCH07072 = 530.67±55.68, muSCH = 7,646.67±233.90). Data bars show mean ± standard error. *** indicate significant differences from HeLa cells at *p<0.001* (one-way analysis of variance and Tukey's post hoc *t*-test). **C**. Western blot analysis using an anti-HSV DNA polymerase antibody demonstrated that less HSV DNA polymerase protein was present in SCH07072 cells compared to wild type HeLa cells 48 hr after virus challenge.

GFP expression levels in the four cell lines challenged with three different MOI (0.1, 0.5, and 1.0) of HSV::GFP were observed under a fluorescence microscope 48 hours after virus challenge. The SCH07072 line showed the lowest GFP expression compared to that of the other two lines (SCH07041 and SCH07071) and the mutant line (muSCH) (**Figure 2A**). The plaque assay revealed that SCH07072 cells produced the fewest number of viral progeny of 163/ml at an MOI of 0.1 and 530/ml at an MOI of 0.5. The plaque assay data supported the GFP expression results showing the best antiviral effects among the three cell lines. In contrast to SCH07072, wild-type HeLa cells had 1,343/ml (8.24 times higher) and 7,851/ml (14.81 times higher) viral progeny at MOIs of 0.1 and 0.5, respectively (**Figure 2B**). At an MOI of 0.5, muSCH cells produced a viral titer that was 14.42 times higher than that of SCH07072 and was similar to that of wild type HeLa cells (**Figure 2B**). Western blot analysis using an anti-HSV DNA polymerase antibody (POL/UL42 complex) demonstrated that less HSV-1 DNA polymerase was detected in SCH07072 cells compared to that of wild-type HeLa cell lines 48 hours after virus challenge (**Figure 2C**).

**Figure 3:**
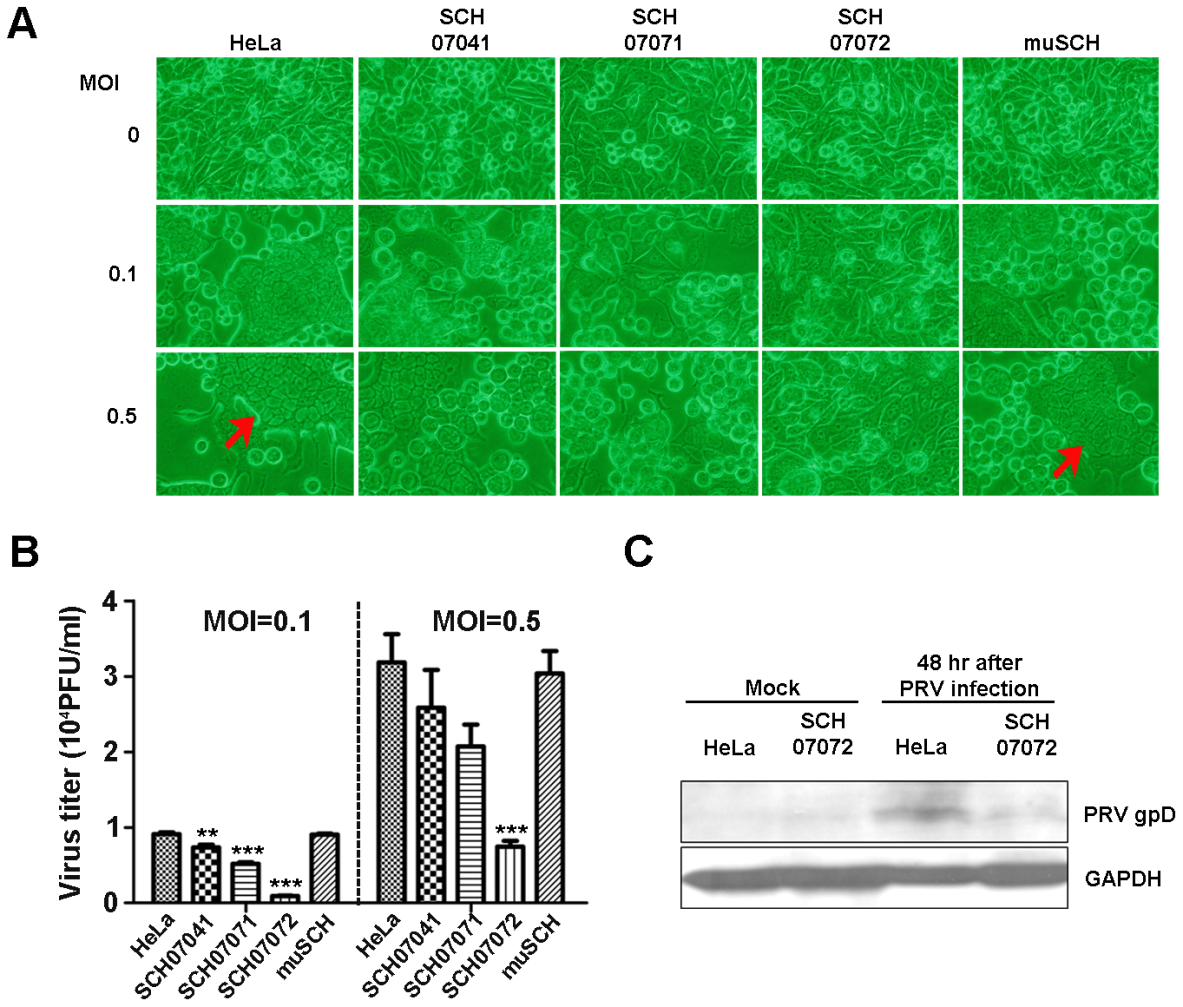
3D8 scFv expression in transgenic HeLa cells confers resistance to PRV. **A**. Three 3D8 scFv cell lines and one mutant 3D8 scFv cell line were challenged with PRV at two different MOIs (0.1 and 0.5). The multinuclear giant cell formation was less frequent in the SCH07072 lines than that in the other cell lines including wild type cells. Red arrow points to CPE (multinuclear giant cell formation). **B**. Wild-type HeLa, SCH07072, and muSCH cells were infected with PRV at 0.1 and 0.5 MOI. Plaque assay data showed the highest antiviral effects in SCH07072 cells (0.1 MOI: HeLa = 9,130.00±222.79, SCH07041 = 7,340.00±389.74, SCH07071 = 5,190.00±182.30, SCH07072 = 916.67±52.07, muSCH = 9,046.67±145.18; 0.5 MOI: HeLa = 31,833.33±3,755.14, SCH07041 = 25,866.67±4,969.35, SCH07071 = 20,700.00±2,902.30, SCH07072 = 7,466.67±762.31, muSCH = 30,400.00±2,959.73). Data bars show mean ± standard error. **, *** indicate significant differences from HeLa cells at *p<0.01* and *p<0.001*, respectively (one-way analysis of variance and Tukey's post hoc *t*-test). **C**. Western blot analysis using an anti-PRV gpD antibody demonstrated that less PRV gpD was present in SCH07072 cells 48 hr after virus challenge than that of wild type HeLa cells.

Similar to HSV, PRV also showed similar antiviral effects in the three 3D8 scFv cell lines and one mutant cell line. Forty-eight hours after the PRV challenge, fewer cytopathic effects, as assessed by multinuclear giant cell formation, were observed in SCH07072 cells compared to the other types of cells, including wild-type cells (**Figure 3A**). The plaque assay also showed the highest antiviral effects in SCH07072 cells with viral titers of 916/ml and 7,466/ml at MOIs of 0.1 and 0.5, respectively, compared to those of wild-type HeLa cells (9,130/ml and 31,833/ml, respectively) and the muSCH cell line (9.046/ml and 30,400/ml, respectively (**Figure 3B**). The expression levels of 3D8 scFv in SCH07071 and SCH07072 cells were similar (**Figure 1B**) but the antiviral activity of SCH07072 and SCH07071 were quite different. Generally there is a correlation between 3D8 scFv expression and antiviral activity. However, in order for cells to acquire the antiviral activity of 3D8 scFv, a certain amount of 3D8 scFv protein needs to be expressed. We thought that there might be a threshold for the acquired antiviral activity conferred by 3D8 scFv expression. SCH07072 may have reached the threshold but SCH07071 did not (**Figure 3B**). Glycoprotein D (gpD) was used for Western analysis to count the PRV. The gpD protein was not found in the SCH07072 lines but wild-type HeLa cells showed strong signals (**Figure 3C**). Even though the expression level of 3D8 scFv in SCH07041 cells was significantly lower (approx. 1/3) than that of SCH07072 cells, both cells were found to be equally resistant against HSV-1 infection at an MOI of 0.1 (**Figure 2B**), suggesting that the 3D8 scFv protein level in SCH07041 cells was close to the maximum effective dose against HSV-1. At a higher virus titer (MOI 0.5), the antiviral effect of 3D8 scFv was shown in a more dose-dependent manner such that much less viral infectivity was found in the SCH07072 cells than the SCH07041 (1,964.00 vs 530.67 pfu/ml). This was further demonstrated in case of PRV infection study (**Figure 3B**), in which the SCH07072 cells were shown to be more resistant against the virus infection than the SCH07041 cells at both MOI of 0.1 and 0.5. As a result, 3D8 scFv expression levels and antiviral effects showed a directly proportional relationship.

### SCH07072 have RNase and DNase activity

**Figure 4:**
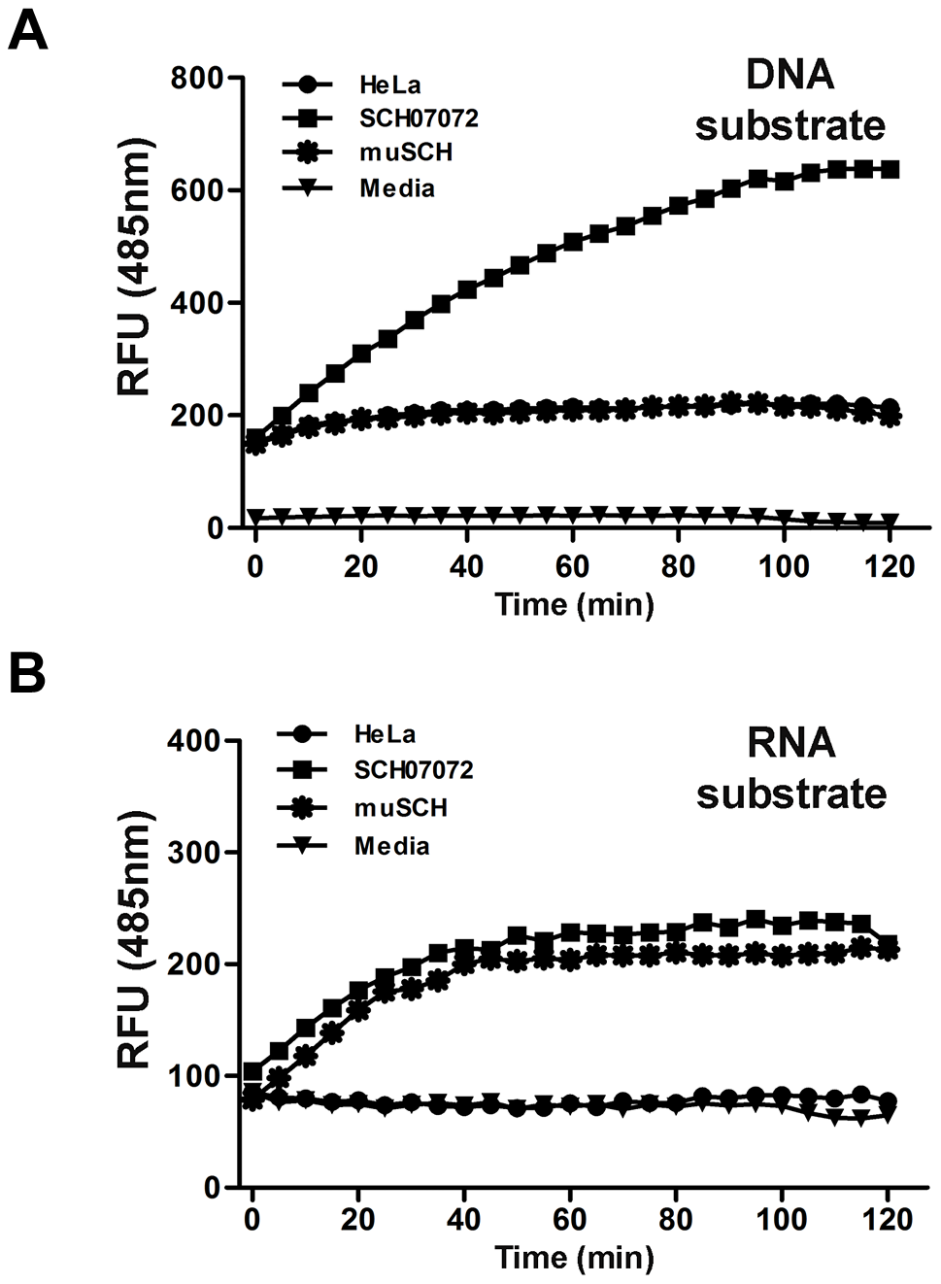
3D8 scFv protein have DNase and RNase activity *in vitro* (FRET assay). **A**. DNA substrate (dsDNA) labeled with 6-carboxyfluorescein (FAM) was transferred into the different cell lines using Lipofectamine. RFUs were determined by measuring the absorbance at 485 nm. Background fluorescence was measured in wells with medium only. SCH07072 cells produced fluorescence counts up to 660 RFU 80 min after treatment. **B**. RNA substrate (ssRNA) labeled with 6-carboxyfluorescein (FAM) was transferred into the different cell lines using Lipofectamine. Fluorescence was determined by measuring absorbance at 485 nm. Background fluorescence was measured in wells with medium only. SCH07072 and muSCH had fluorescence levels of approximately 200 RFU in contrast to the fluorescence counts of wild-type HeLa cells and the negative control reactant, which were approximately 80 RFU.

The FRET assay was carried out to confirm that 3D8 scFv nuclease activity was responsible for the antiviral activity shown by SCH07072 cells [32] (**Figure 4**). SCH07072 cells produced up to 660 relative fluorescence units (RFU) 80 min after the 6-carboxyfluorescein (FAM)-labeled DNA substrate was introduced into the cells. However, the fluorescence output from HeLa cells, muSCH cells, and the negative control reactant was a maximum of 200 RFU or less (**Figure 4A**). When the FAM-labeled RNA substrate was introduced into cells, SCH07072 and muSCH cells had fluorescence levels of about 200 RFU, but HeLa cells and the negative control reactant showed fluorescence levels of only 80 RFU (**Figure 4B**). These FRET results confirmed that 3D8 scFv in SCH07072 had both DNase and RNase activity, but that only RNase activity was observed in muSCH cells (**Figure 4**). Therefore, the protection against the virus infection shown by SCH07072 cells was due to 3D8 scFv nuclease activity against the virus genome itself.

### 3D8 scFv expressing cells induce viral DNA degradation in the nucleus and viral mRNA degradation in the cytosol

**Figure 5:**
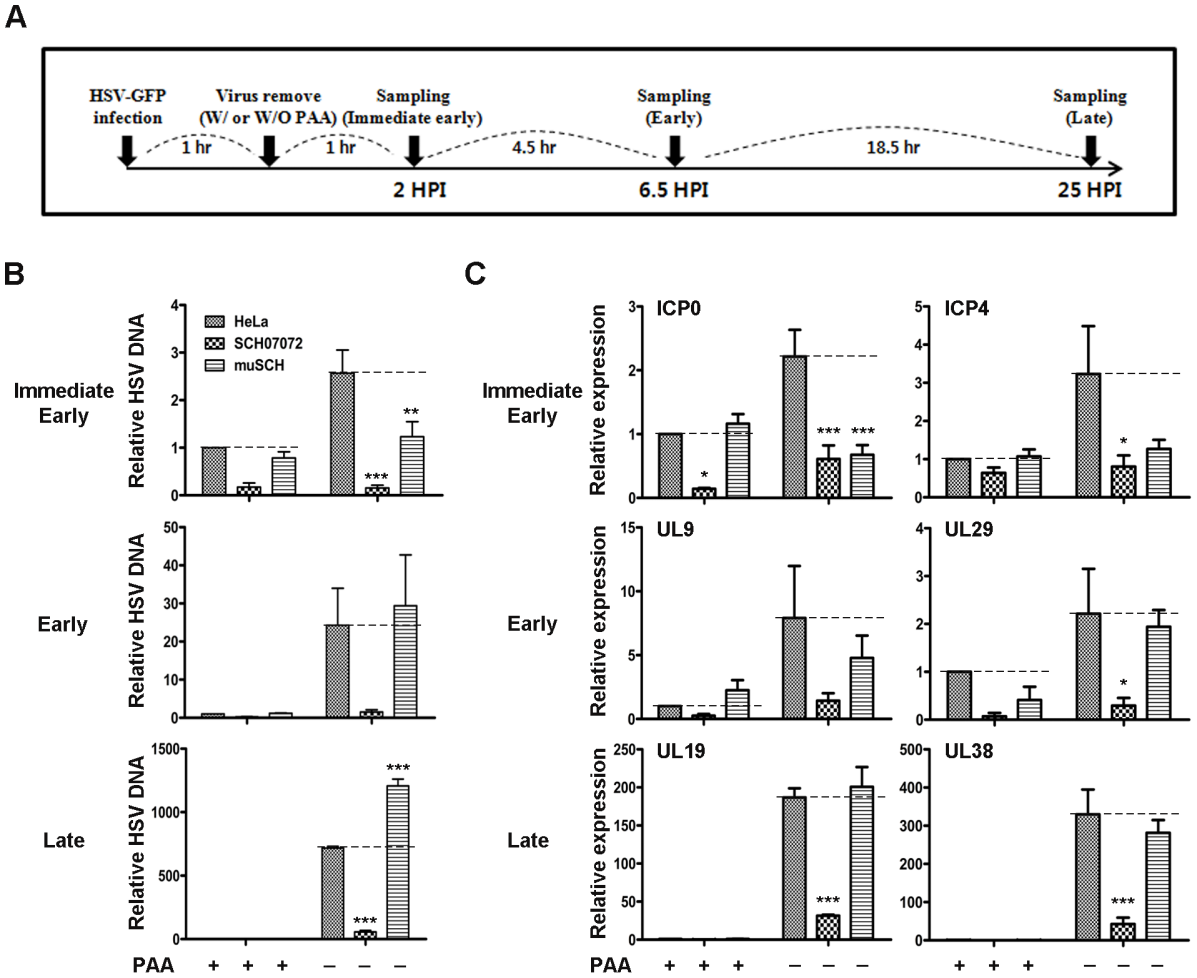
3D8 scFv inhibits HSV-1 encoded gene expression by DNase and RNase activity. **A**. Schematic diagram for identification of viral gene expression pattern by HSV-1 challenging. **B**. Wild-type HeLa, SCH07072, and muSCH cells were infected with HSV::GFV at an MOI of 0.5 and then incubated for 2, 6.5, and 25 hr in the presence or absence of PAA 1 hr after virus challenge. ICP4 was used for the immediate early stage, UL9 for the early stage, and UL19 for the late stage of viral infection. Data bars show mean ± standard error. ** and *** indicate significant differences from HSV-1 viral DNA at *p<0.01* and *p<0.001*, respectively (one-way analysis of variance and Tukey's post hoc *t*-test). **C**. Quantitative real-time reverse transcription-polymerase chain reaction (RT-PCR) was used to measure the expression of immediate early genes (ICP0 and ICP4), early genes (UL9 and UL29), and late genes (UL19 and UL38). The relative concentrations of HSV mRNAs were calculated after normalization to the GAPDH gene using the delta delta C_T_ method. Data bars represent mean ± standard error. *, **, *** indicate significant differences from HeLa cells at *p<0.05, p<0.01*, and *p<0.001*, respectively (one-way analysis of variance and Tukey's post hoc *t*-test).

Quantitative RT-PCR was performed to investigate how 3D8 scFv protected against virus infection in relation to the virus infection cycle. The amounts of viral DNA and RNA isolated from cells grown with or without HSV DNA polymerase inhibitor (Phosphonoacetic acid, PAA) in the culture media were analyzed by quantitative real-time PCR (**Figure 5**). PAA inhibited the synthesis of HSV DNA in infected cells and the activity of the virus-specific DNA polymerase *in vitro* [33]. PAA was used to investigate the antiviral effects by 3D8 scFv DNase activity in the immediate early stage before *de novo* viral DNA replication. The changes in viral DNA content in HeLa, SCH07072, and muSCH cells were traced at immediate early (2 hr after virus challenge, ICP4), early (6.5 hr after virus challenge, UL9), and late (25 hr after virus challenge, UL19) stages of infection (**Figure 5A**). Viral DNA accumulation in SCH07072 cells was reduced by up to 94.2% under the PAA-untreated condition, compared to HeLa cells at each stage. Virus DNA accumulation in SCH07072 cells cultured with PAA decreased by up to 80% compared to that in HeLa cells at all stages of infection. In contrast, muSCH cells under the PAA-untreated condition showed as much virus DNA accumulation as HeLa cells at the early stage of infection, but 68% more viral DNA was detected in muSCH cells compared to HeLa cells at the late stage. The accumulation of virus DNA in SCH07072 indicates that nuclear 3D8 scFv can degrade viral DNA directly, resulting in less accumulation of viral DNA (**Figure 5B**).

Viral RNA expression was also analyzed under the same experimental conditions. Viral RNA accumulation of six ORFs decreased rapidly in PAA-treated cells at both the early and late stages of infection (**Figure 5C**). Only SCH07072 cells showed a substantial reduction in viral RNA accumulation during the entire infection cycle under the PAA-untreated condition compared to HeLa and muSCH cells. The expression of ICP0 and ICP4 in SCH07072 cells cultured with PAA decreased by 72.5% and 74.9%, respectively, compared to HeLa cells during the immediate early stage. At the late infection stage, SCH07072 cells expressed 83.1% and 87% less UL19 and UL 38 transcripts, respectively, than those of HeLa cells after virus challenge (**Figure 5C**). However, ICP0 and ICP4 levels were 69.4% and 60.7% lower than in HeLa cells during the immediate early stage of muSCH cell infection, and were similar to the viral RNA content seen in SCH07072 cells. However, at the late stage of infection, the viral RNA accumulation patterns in muSCH cells were similar to those in HeLa cells (**Figure 5C**).

The HSV-1 RNA transcription occurs during immediate early stage of the viral infection stage together with a limited level of its DNA replication, as summarized on Edward K. Wagner's web site (http://darwin.bio.uci.edu/~faculty/wagner/hsv4f.html). Therefore, in the absence of PAA the decreased level of viral DNA in muSCH cells in comparison with that of HeLa cells is likely to be attributed to an interference of HSV -1 transcription by the RNase activity of the mutant 3D8 scFv (**Figure 5B**). With treatment of PAA, a specific inhibitor of viral DNA polymerase, the level of viral DNA replication in muSCH and HeLa cells were shown to be almost identical at immediate early stage. This relatively mild impact of PAA on DNA replication in muSCH cells as compared with HeLa and SCH07072 cells was also observed when selected viral RNA transcripts were measured in these cells at immediate early stage (**Figure 5C**). Transcriptions of these genes were found to be significantly decreased in HeLa and SCH07072 cells upon PAA, which reflects the decreased level of viral DNA templates for transcription. By contrast, transcription level of these genes in muSCH cells at the same stage did not show any significant difference between PAA treated and untreated cells.

On entry into the nucleus, the genome of HSV-1 is also associated rapidly with histone proteins [34] and nucleosomes are assembled, though in irregularly or randomly spaced manners, on 1 hour post infection. Therefore, it is intriguing and difficult to explain on why and how only the viral genome is subjected to the nucleic acid hydrolyzing activity of 3D8 scFv, leaving host genome largely unaffected. Perhaps as the HSV-1 genome, which was not completely assembled with nucleosomes or was assembled with unstable nucleosomes, can be digested by intrinsic DNase of 3D8 scFv during HSV-1 replication takes place (6 hours post infection or early stage), especially considering that the viral genome is found to be relatively free of histones following viral DNA replication, beyond 6 hours post infection [35]. When we compared the HSV-1 DNA accumulation between SCH07072 and muSCH cells on immediate early stage, early stage and late stage respectively, muSCH cells showed limited antiviral activities at immediate early stage but did not show any antiviral activity later on. Thus it may indicates that mutant 3D8 scFv with only intrinsic RNase activity is not sufficient for providing a full protection for host cells against HSV-1 infection once HSV-1 DNA replication successfully takes places in nucleus in muSCH cells. Therefore, it may be possible that the antiviral effects of 3D8 scFv against HSV-1 and PRV infection were contributed by both the DNase activity, which is mainly effective in the nucleus and the RNase activity mainly effective in the cytoplasm. This proposed antiviral activity can be explained by (1) temporal blockage of viral DNA replication in the nucleus and protein translation in the cytosol and (2) spatial protection (nucleus vs. cytosol) provided by 3D8 scFv. However, further experimental investigations are needed to confirm the antiviral mechanisms of 3D8 scFv proposed in this study.

### 3D8 scFv protein can distinguish methylated DNA and histone-bound DNA

**Figure 6:**
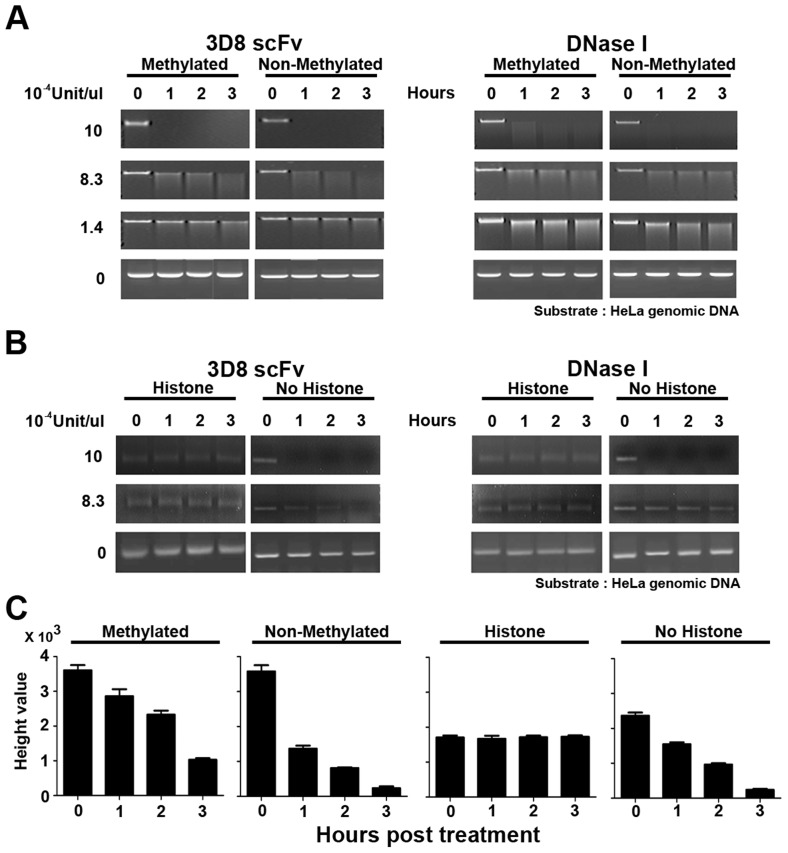
3D8 scFv cannot hydrolyze methylated DNA or protein-bound DNA. **A**. HeLa methylated genomic DNA and non-methylated DNA (NEB) were treated with 3D8 scFv and DNase I at four concentrations: 10×10^−4^ U/µl, 8.3×10^−4^ U/µl, 1.4×10^−4^ U/µl, and 0 U/µl. The DNA samples were harvested and analyzed by electrophoresis at 0, 1, 2, and 3 hr after treatment. At a concentration of 8.3×10^−4^ U/µl, 3D8 scFv digested more non-methylated DNA than DNase I 1 hr after treatment. **B**. Chromatin was prepared using the EZ-Zyme Enzymatic Chromatin Prep kit. Prepared chromatin DNA and naked DNA were treated with 3D8 scFv and DNase I (10×10^−4^ U, 8.3×10^−4^ U, and 0 U/µl). The DNA samples were harvested and analyzed by electrophoresis at 0, 1, 2, and 3 hr after treatment. Neither 3D8 scFv nor DNase I hydrolyzed histone-bound DNA at concentrations of 10×10^−4^ U/µl and 8.3×10^−4^ U/µl. When naked DNA was treated with 3D8 scFv at concentrations of 8.3×10^−4^ U/µl, 3D8 scFv could digest naked DNA. But, DNase I could not digest naked DNA at 8.3×10^−4^ U/µl. **C**. The relative quantitative values of each DNA of methylated, non-methylated, histone-bound, and naked HeLa genomic DNA were measured for 3 hr after each DNA was treated with 3D8 scFv (8.3×10^−4^ U/µl). The bar graphs indicate the average values of each sample calculated from three individual experiments.

The 3D8 scFv protein shown in SCH07072 was localized in both the cytoplasm and nucleus (**Figure 1D**). To test our hypothesis that 3D8 scFv acted as a DNase in the nucleus, we investigated whether 3D8 scFv could hydrolyze histone-bound DNA and methylated DNA. Both 3D8 scFv and DNase I at 10×10^−4^ U/µl started to hydrolyze 0.2 µg DNA 1 hr after treatment. At a concentration of 8.3×10^−4^ U/µl, 3D8 scFv digested more non-methylated DNA than methylated DNA after 1 hr of treatment (**Figure 6A**). In addition, quantitation of non-methylated and methylated DNA showed a reduction rate of 62% and 21%, respectively (**Figure 6C**). However, no difference in hydrolysis of non-methylated and methylated DNA was observed when 8.3×10^−4^ U/µl DNase I was tested (**Figure 6A**). Both 3D8 scFv and DNase I did not digest DNA at a concentration of 1.4×10^−4^ U/µl, regardless of whether it was methylated or not (**Figure 6A**). We next tested whether the 3D8 scFv protein could digest histone-bound DNA in the nucleus. Histone-bound DNA fragments (150 bp) were not hydrolyzed by either 3D8 scFv or DNase I at concentrations of 10×10^−4^ U/µl and 8.3×10^−4^ U/µl, as shown in Figure 6B. But, at concentrations of 8.3×10^−4^ U/µl 3D8 scFv, DNA without histones was reduced 34% and 90% after 1 hr and 3 hr, respectively (**Figure 6C**). Also, 3D8 scFv digested more DNA without histones than DNase I at concentrations of 8.3×10^−4^ U/µl 1 hr after treatment (**Figure 6B**).

The nuclear DNA of eukaryotic cells is mostly modified by methylation and interacts with various nuclear proteins such as histone proteins [36], [37], [38], [39]. Figure 6 shows that 3D8 scFv did not hydrolyze methylated or histone-bound DNA when the activity of 3D8 scFv was <1.4×10^−4^ U/µl. This result indicates that chromosomal DNA in the nucleus was protected from 3D8 scFv because chromosomal DNA is mostly methylated and histone-bound., 3D8 scFv proteins in SCH07072 cells are expressed at lower working concentrations than we tested in our *in vitro* analysis shown in Figure 6.

HeLa, SCH07072, and muSCH cells showed similar doubling patterns when 72 hr growth curves were plotted (**Figure S1A**). Northern blot analysis showed that the RNA stability and RNA expression patterns of three marker genes (GAPDH, actin, and VEGF) in the three cell lines were not affected by expression of the 3D8 scFv protein (**Figure S1B**). Taken together, expression of the 3D8 scFv protein in SCH07072 cells did not affect cell growth or viability.

Pac1 and ISG20, which have RNase activity, have been used as antiviral proteins against potato spindle tuber viroid and HIV, respectively [21], [22]. Our observations and those of others on the effects of exogenous expression of RNase (Pac1 and ISG20) and/or nuclease (3D8 scFv) indicate that it is possible to develop transgenic plants and animal cells and even entire organisms that are virus-resistant. However, host RNA could be degraded non-selectively by 3D8 scFv RNase activity in 3D8 scFv-expressing cells. During the transformation process, antiviral transgenic cell lines or organisms can be developed if (1) the amount of 3D8 scFv protein expressed does not inhibit the physiological and developmental processes of cells or organisms, and (2) the amount of 3D8 scFv protein produced is sufficient to protect against virus infection.

3D8 scFv and DNase I were transferred to HeLa cells using a microporator to investigate cell viability using a neutral red assay. The concentration of each protein was adjusted to be 1.5625×10^−3^ to 0.05 units. No cell viability differences were observed between 3D8 scFv and DNase I for 3D8 scFv and DNase I concentrations of up to 0.05 units (**Figure S2A**). 3D8 scFv and DNase I were detected in both the cytosol and nucleus of HeLa cells under confocal microscopy (**Figure S2B**). Montandon et al. (1982) reported the antiviral effects of DNase I against M-MuLV in mouse cells. DNase I digested non-methylated DNA of proviral M-MuLV but did not hydrolyze methylated endogenous M-MuLV [19]. However, when we introduced the same unit amounts of DNase I and 3D8 scFv into HeLa cells using a microporator, the antiviral effects of the 3D8 scFv protein were almost 8-fold higher than those of DNase I (**Figure S2C**). This result indicates that 3D8 scFv has a greater antiviral effect than DNase I, most likely due to the additional RNase activity of 3D8 scFv (**Figure S2**).

### Production and establishment of 3D8 scFv TG mice and 3D8 scFv gene expression

**Figure 7:**
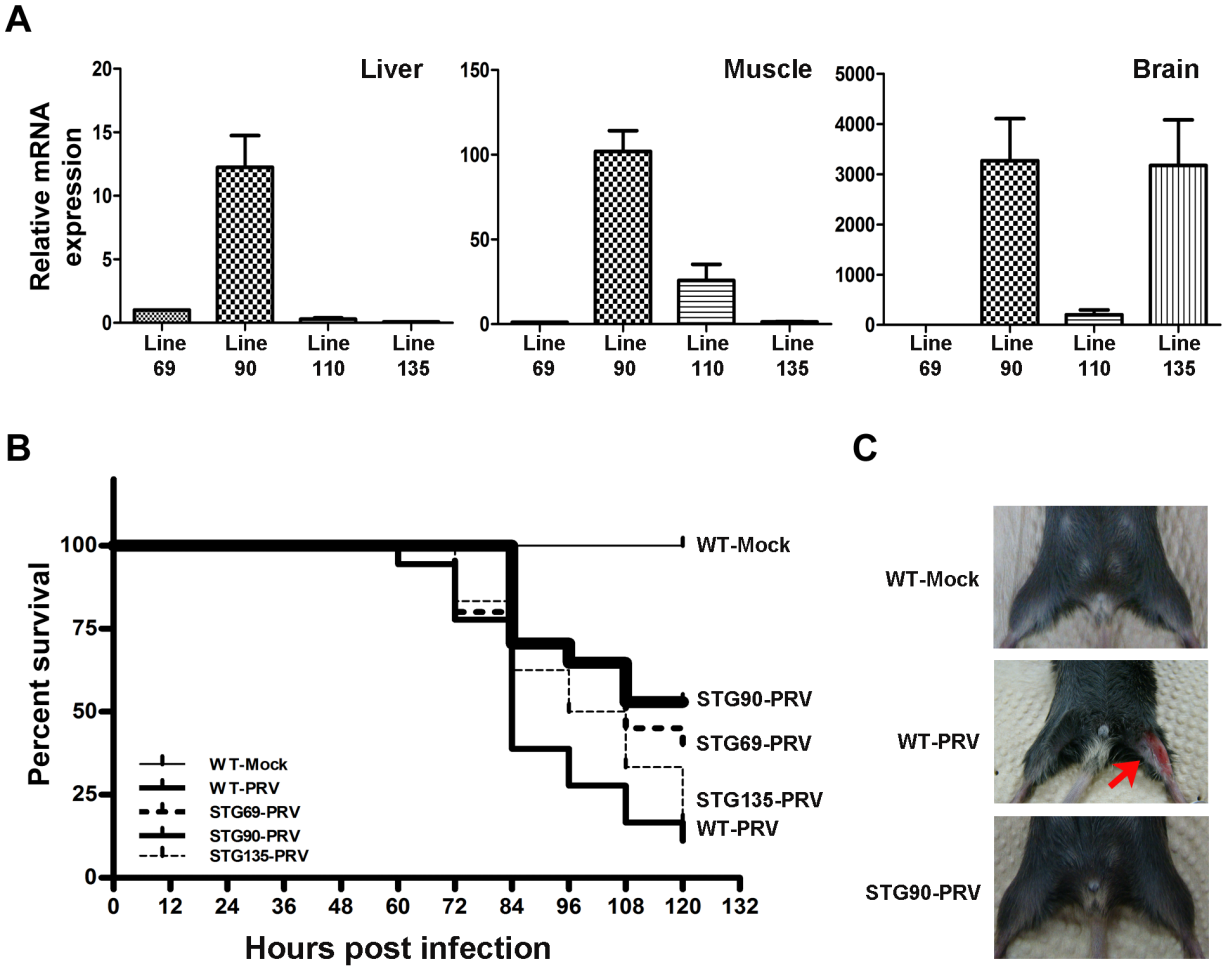
High expression of 3D8 scFv in the muscle and brain of STG90 mice. **A**. 3D8 scFv mRNA expression was verified by quantitative real-time reverse transcription polymerase chain reaction (RT-PCR) analysis in the muscle and brain of TG mice. The STG90 line expressed high levels of 3D8 scFv in both the brain and muscle, whereas the STG135 line expressed high amounts of 3D8 scFv in the brain; these strains were selected for further antiviral preventive studies. The relative expression of 3D8 scFv mRNAs was calculated after normalization to the GAPDH gene using the delta delta C_T_ method. The primer efficiency of 3D8 scFv and GAPDH are 1.845 and 2.065, respectively. Each qRT-PCR data point is a representative example of data from three replicate experiments. Data bars represent mean ± standard error. **B**. Kaplan-Meier survival analysis for antiviral effects of all groups. The difference in survival between WT-PRV and STG90-PRV was statistically significant (p = 0.0042 by log-rank test). The numbers of live and dead mice were counted every 12 hr for 5 days after challenge with 10 LD_50_ PRV in the femoral muscle. Survival was highest in the STG90-PRV group (53%) compared to that of the other groups (WT-PRV: 11%, STG69: 40%, and STG135: 17%). **C**. PRV-challenged F_2_ mice (WT-PRV) began to exhibit symptoms of inflammation in the femoral muscle and finally died 3–5 days post-virus challenge. Red arrow points to inflammation symptoms.

After the antiviral effects of the 3D8 scFv protein was confirmed in HeLa cells, transformed mice harboring the 3D8 scFv gene were produced to investigate the antiviral effects in an *in vivo* mouse system. The pcDNA3.1-3D8 scFv plasmid was linearized with *Nru*I/*Stu*I/*Pvu*I enzymes and then used for transformation of C57BL/6NCrjBgi. A total of 150 F_0_ were generated and 10 lines (F_0_: 47, 69, 90, 92, 108, 109, 110, 115, 128, and 135) out of 150 F_0_ were selected by PCR analysis (**Figure S3A**). Each transformant was reconfirmed by Southern hybridization after genomic DNA was digested by *Eco*RI and *Hind*III enzymes. All transformants showed single bands that reacted with the probes prepared with the 3D8 scFv full gene (**Figure S3B**). 3D8 scFv expression levels were analyzed in the different transgenic lines and different organs. The muscle, brain, and liver were chosen because these organs are virus infection routes and the relative 3D8 scFv gene expression levels were investigated by quantitative real-time RT-PCR (**Figure 7A**). The F_0_ 69, 90, and 135 lines were selected from 10 F_0_ lines for further analysis based on 3D8 scFv expression levels in the TG mice lines and previous therapeutics experiments, which showed that PRV was detected in muscle and brains after mice were challenged with PRV and 3D8 scFv proteins by intramuscular and intraperitoneal injections, respectively. Therefore, the F_0_ 90 line, which expressed the highest 3D8 scFv levels in both brain and muscle, and the F_0_ 135 line, which expressed high amounts of 3D8 scFv in the brain, were selected and used for further investigations on the antiviral preventive effects. The F_0_ 69 line showed positive gPCR and RT-PCR results but 3D8 scFv expression levels in the F_0_ 69 line were low; thus, it was used as a control.

On the basis of the expression levels in the F_0_ 69 line, the F_0_ 90 line produced 12.25 times more 3D8 scFv in the liver and the F_0_ 110 and F_0_ 135 lines showed 0.29 and 0.07 times less than that of the F_0_ 69 line. However, the F_0_ 90 and F_0_ 135 lines had 3,270 and 3,174 times more 3D8 scFv in the brain than that of the F_0_ 69 line, even 201.98 times more in F_0_ 110. Therefore, the F_0_ 69 line was used as a control and the F_0_ 90 and F_0_ 135 lines were used for testing the antiviral preventive effects in TG mice lines after PRV infection (**Figure 7A**).

The 3D8 scFv TG founder (F_0_ lines) was mated with wild-type C57BL/6NCrjBgi to establish the lines and produce siblings from the F_0_ lines. Four F_1_ lines (69 F_1_: 31, 33, 38, 208) were produced from the F_0_ 69 lines. Each of the four F_1_69 lines were mated with wild-type C57BL/6NCrjBgi and then 20 F_2_69 lines were produced. They were used for virus challenge experiments and named STG69. In the case of the 90 and 135 F_1_ lines, three F_1_90 lines (F_1_90: 45, 46, and 162) and five F_1_135 lines (F_1_135: 140, 142, 144, 165, and 166) were produced and then 17 F_2_90 lines (STG90) and 24 F_2_135 lines (STG135) were prepared (**Figure S4A**). Genomic PCR was performed to confirm the transgenic lines at each generation from F_0_ to F_2_ (**data not shown**).

### 3D8 scFv protein has antiviral preventive effects

The number of live and dead mice was counted every 12 hr for 5 days to investigate the viability in each TG line after the femoral muscle was challenged with 10 LD_50_ PRV was (**Figure S4B**).

Figure 7B shows that the viability of WT mice challenged with PRV was 11% (2/18). The viabilities of the STG69 and STG135 lines against PRV challenges were about 40% (8/20) and 17% (4/24), respectively. However, the STG90 line showed 53% (9/17) viability. These data were described by Kaplan–Meier analysis. Mice generally began to exhibit clinical signs of illness 3–5 days post-challenge (**Figure 7C**).

**Figure 8:**
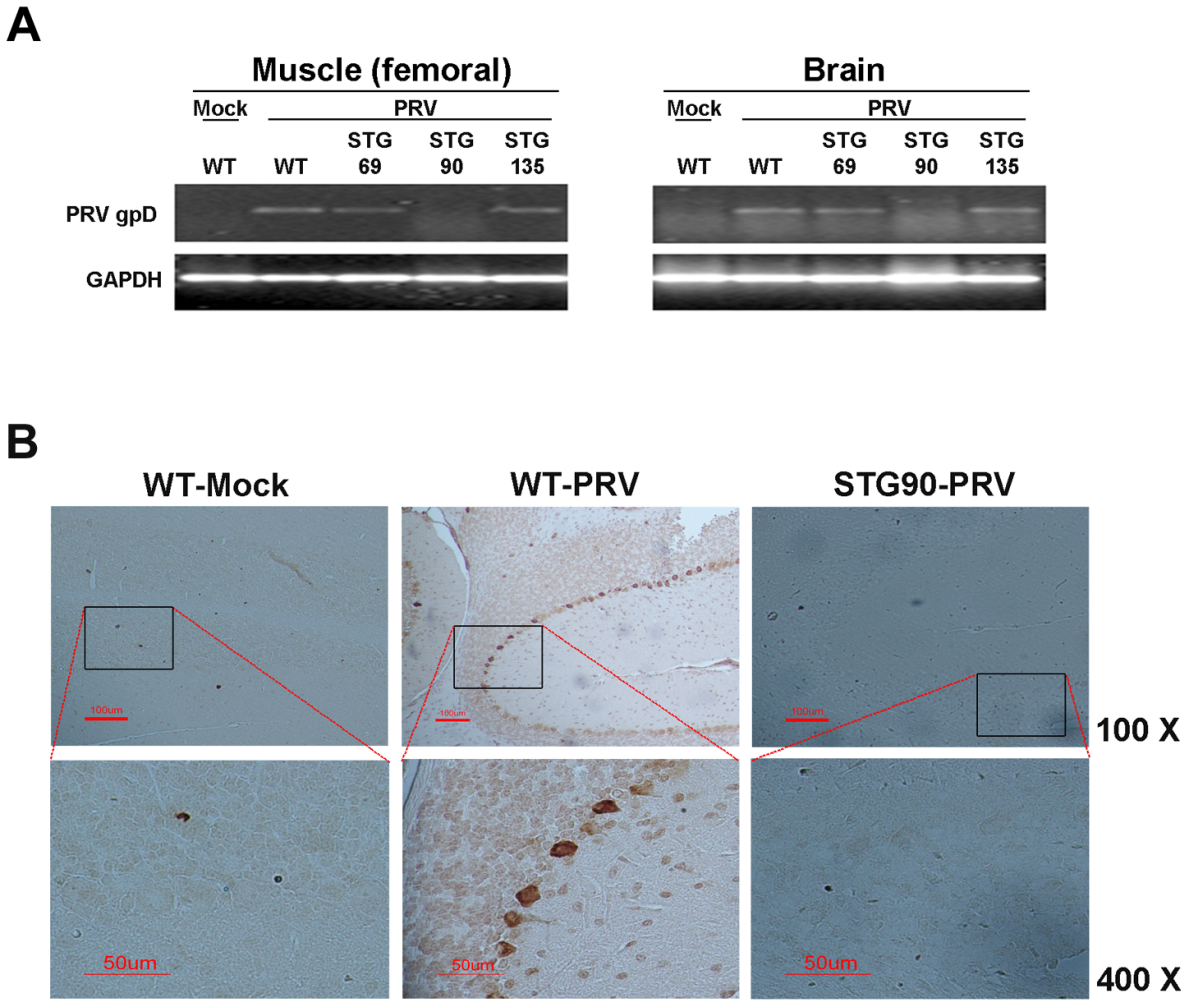
STG90 exhibits antiviral effects against PRV. **A**. The expression levels of PRV gpD RNA in the muscle and brain of WT-Mock, WT-PRV, STG69-PRV, STG90-PRV, and STG135-PRV mice were investigated. Only live STG90-PRV mice did not show PRV gpD expression. **B**. Immunohistochemistry was performed to detect PRV in PRV target organs; brain (upper panel: ×100) and femoral muscle (bottom panel: ×400). The PRV gpD protein was stained with a monoclonal anti-PRV antibody and visualized with DAB. The Purkinje layer cells in the WT-PRV group stained brown for the gpD protein, whereas no staining was observed in the other groups.

RT-PCR and immunohistochemistry were performed to confirm virus accumulation in the three 3D8 scFv TG lines after PRV inoculation. Figure 8A shows that PRV gpD RNA was detected in the muscle and brain of WT, STG69, and STG135 mice accompanied by disease symptoms. Immunohistochemical analysis with an anti-gpD antibody supported the viral gene expression data; only WT mice with a high infection rate had Purkinje layer cells from the brain that stained dark brown after diaminobenzidine (DAB) staining (**Figure 8B**).

These data indicate that virus multiplication was not inhibited by the 3D8 scFv protein if virus infection was not protected against in the early infection stages by the 3D8 scFv protein. This also indicates that PRV DNA was hydrolyzed immediately in virus-inoculated STG90 muscle cells, and that the virus titer was not high enough to infect muscle cells. Therefore, PRV could not move systemically into the spinal cord or brain in STG90 mice. However, STG135 mice were not protected against PRV infection even though 3D8 scFv protein was expressed in the brain (**Figure 7A**) at similar levels to those observed in STG90 mice. We interpret these data to indicate that the expression and presence of antiviral proteins in inoculated cells and tissues is as important as high expression levels in target cells or tissues. Blood biochemistry (**Table 1**) and body weight changes for 7 weeks after birth (**Figure S5A and S5B**) were not significantly different between STG90 and WT mice. Also, we identified the expression patterns of endogenous genes including apoptosis (K-ras and BAX), growth factor (VEGF), and housekeeping (GAPDH) genes between STG90 and wild-type mice; however, there was no difference (**Figure S5C**).

**Table 1:** Blood chemistry analysis (mean ± standard error).

Group		ALB (g/dL)	ALT (unit/L)	AST (unit/L)	ALP (unit/L)	Glu (g/dL)	TG (mg/dL)	BUN (mg/dL)
Female	WT	4.6±0.1	44.7±2.9	109.2±6.4	528.0±46.5	218.0±21.7	51.0±3.0	17.0±0.8
	STG90	4.6±0.2	40.0±2.0	103.0±15.6	741.0±41.0	227.0±18.7	46.0±16.8	23.4±2.6
Male	WT	4.0±0.1	42.0±4.6	68.0±2.6	535.0±88.6	269.0±25.1	31.0±8.2	20.8±1.1
	STG90	4.2±0.0	46.0±4.0	72.0±10.4	663.0±51.5	242.0±5.6	32.0±1.0	19.2±2.9

Standard blood biochemistry parameters were analyzed in 7-week-old WT C57BL/6 and STG90 mice (n = 9 mice per genotype and gender). ALB, albumin; ALT, alanine transaminase; AST, aspartate aminotransferase; ALP, alkaline phosphatase; Glu, glucose; TG, triglycerides; CK, creatine phosphokinase; BUN, blood urea nitrogen.

Although we have demonstrated the antiviral effect attributed by DNase and RNase activity of 3D8 scFv without any cytotoxic effect on the host cells in this study (**Figure S1A and S1B**), possibility of the host DNAs damaged by non-specific nuclease activity of 3D8 scFv cannot be entirely ruled out. In our previous study, when we tested the potential cytotoxic effect of 3D8 scFv on HeLa cells with varying concentration of 3D8 scFv (5–40 µM) for 48 hrs, the cell viability was found to drop by ∼50–60% upon 10 µM of 3D8 scFv treatment, while treatment of the cells with 1 µM and 5 µM 3D8 scFv exhibited no significant cytotoxicity up to 48 h of incubation (0 and ∼20% respectively) [40]. This may indicate that the host cells indeed begin to be affected by the non-specific activity of 3D8 scFv at higher dosages (i.e., 5 µM and up). Although it is not clear on exactly how much 3D8 scFv protein actually penetrated into the cells from media when 5 µM 3D8 scFv protein was administered, 5 µM 3D8 scFv is fairly large amount of proteins for in vitro cytotoxicity analysis even though we used the same amount of the proteins for in vivo survival experiments with mice (**Figure 7**). On the other hand, the actual level of 3D8 scFv expressed in transgenic cells such as in SCH07072 cell line was too low to be even detected by Western blot analysis, although its expression was confirmed by confocal microscopic observation and flow cytometry analysis (**Figure 1**). Therefore, it can be concluded that the low concentration of 3D8 scFv expressed in transgenic cell lines or TG mice is generally sufficient for conferring antiviral effects without incurring damage to the host DNA, but the likelihood of 3D8 scFv having deleterious impact on host cells as well through non-specific nucleic acid hydrolyzing activity remains valid, especially at higher concentration of the protein.

**Figure 9:**
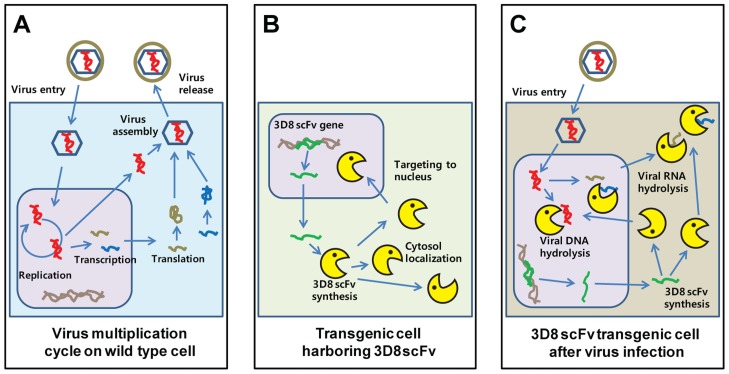
New antiviral mechanism by 3D8 scFv protein. **A**. Model of the HSV-I replication cycle. Virus infection begins with binding of the virus to the cell surface. The viral envelope fuses with the cell membrane and delivers the viral capsid into the cytoplasm. Viral DNA synthesis begins shortly after the appearance of the beta proteins and the temporal program of viral gene expression ends with the appearance of the gamma or late proteins, which constitute the structural proteins of the virus. Finally, the virus undergoes a lytic cycle. **B**. Expression model of 3D8 scFv proteins. 3D8 scFv proteins were localized in cytosol using a vector system and targeted in the nucleus by nuclear localization signal. Therefore, 3D8 scFv proteins in SCH07072 were present in both the cytosol and nucleus. **C**. 3D8 scFv has a unique dual and stereoscopic protection mechanism that includes DNase activity in the nucleus and RNase activity in the cytoplasm. 3D8 scFv acts by inhibiting (1) viral DNA replication and RNA transcription in the nucleus via viral DNA degradation and (2) translation in the cytoplasm via viral RNA degradation. In other words, 3D8 scFv targets the viral DNA genome itself or its RNA transcripts spatially in two different subcellular spaces (nucleus and cytoplasm) and at two different times.

In conclusion, the DNA virus-protective effects conferred by the 3D8 scFv protein can be attributed to the DNase activity of the protein in the nucleus and RNase activity in the cytoplasm. This antibody inhibits (1) viral DNA replication and RNA transcription in the nucleus by viral DNA degradation and (2) viral protein translation in the cytoplasm via viral RNA degradation. In other words, the 3D8 scFv protein attacks the virus DNA genome itself or its RNA transcripts in two different subcellular spaces (nucleus and cytoplasm) and at two different times (viral replication and viral transcription) (**Figure 9**). These antiviral effects of 3D8 scFv have not only been observed against viruses with a dsDNA genome but also ssRNA viruses such as CSFV [25] and several plant viruses such as geminivirus, tobamovirus, and cucumovirus [26], [27]. Taken together, 3D8 scFv is a candidate antiviral protein that can potentially confer resistance to a broad spectrum of animal and plant viruses.

## Materials and Methods

### Cells and viruses

HeLa cells were provided by the Korean Cell Line Bank and were maintained in DMEM medium supplemented with 10% fetal bovine serum (Hyclone, Logan, UT, USA), 100 U/ml penicillin-streptomycin (Hyclone), and non-essential amino acids (Sigma, St. Louis, MO, USA), at 37°C in a 5% CO_2_ atmosphere. HSV-GFP virus was obtained from the American Type Culture Collection (ATCC Number: VR-1544). The PRV-YS strain was obtained from the National Veterinary Research and Quarantine Service (NVRQS) of Korea.

### Antibodies

Anti-3D8 scFv polyclonal antibody was provided by Dr. Kwon (Ajou University School of Medicine). Anti-PRV gD monoclonal antibody was purchased from Jeno Biotech Inc. (Chuncheon, Korea) and anti-HSV monoclonal antibody was obtained from Chemicon (Temecula, CA, USA). Anti-GAPDH polyclonal antibody was purchased from Santa Cruz Biotechnology (Santa Cruz, CA, USA).

### Establishment of 3D8 scFv- and mu3D8 scFv-expressing HeLa cell lines

3D8 scFv and mu3D8 scFv were amplified by PCR from the pIg20-3D8 scFv vector [23] and then subcloned into the pcDNA3.1 V5/His-B vector (Invitrogen, Carlsbad, CA, USA). pcDNA3.1-3D8 scFv and pcDNA3.1-mu3D8 scFv were transfected into HeLa cells using Fugene HD transfection reagents (Roche, Indianapolis, IN, USA) and selected by G418 selection (400 µg/ml). The relative concentrations of 3D8 scFv and mu3D8 scFv were calculated by quantitative real-time PCR after normalization to the GAPDH gene (Table 2.). The primer efficiency of 3D8 scFv and GAPDH are 1.845 and 2.065, respectively.

**Table 2:** Polymerase chain reaction (PCR) primers for the detection of viral genes and global genes analyzed in this study.

Gene Name	Forward (5′- 3′)	Reverse (5′- 3′)	Accession No.
**hGAPDH**	GTCAGTGGTGGACCTGACCT	CCCTGTTGCTGTAGCCAAAT	NM_002046
**3D8 scFv**	ACTGACTTCAGACAAATCCA	CTGTGACATCACAAGATCTGAGC	AF232220 & AF232221
**PRV gpD**	CGTACCGCGCCCACGTGGCC	GTCGGTGAGGATGTTCACGC	NC_006151
**ICP0**	CACCACGGACGAGGATGAC	CGGCGCCTCTGCGT	NC_001806
**ICP4**	GCAGCAGTACGCCCTGA	TTCTGGAGCCACCCCATG	NC_001806
**UL9**	TAGTTTTTCCCGACCCCATT	ACGAGTGCGAACAGTACACG	NC_001806
**UL29**	CGCTCCAGGTAAAACAGCAT	TTTACCGCTTCTTCCTCGTG	NC_001806
**UL38**	CGGGCCTAGTGTCGTTTAACT	GACACTCGGAAAAACGATCC	NC_001806
**UL19**	CCATCCAAAATGGCGACTAT	AAAGTAGTTGGCCCCCAGAG	NC_001806
**mGAPDH**	ACCCAGAAGACTGTGGATGG	CACATTGGGGGTAGGAACAC	NM_008084
**VEGF**	ACACGGGAGACAATGGGATG	TCTTGACTCAGGGCCAGGAA	NM_001025250
**K-Ras**	TTTGGTGCATGCAGTTGATT	GTGACCCCTCAGTGTCCAGT	NM_021284
**Bax**	TGCAGAGGATGATTGCTGAC	GATCAGCTCGGGCACTTTAG	NM_007527

### Immunocytochemistry and flow cytometry

Confocal microscopy and flow cytometry were performed as described previously [40]. Cells on coverslips were washed in phosphate-buffered saline (PBS) and fixed for 10 min in 4% paraformaldehyde in PBS at room temperature. Cells were permeabilized with Perm-buffer (1% BSA, 0.1% saponin, 0.1% sodium azide in PBS) for 10 min at room temperature (RT). After blocking with 3% BSA in PBS for 1 hr, 3D8 scFv-treated cells were incubated with rabbit anti-3D8 scFv Ab, followed by TRITC-anti-rabbit Ig. Nuclei were stained with DAPI during the last 10 min of incubation at RT. Cells on coverslips were mounted in Vectashield anti-fade mounting medium (Vector Labs, Burlingame, CA, USA), and observed with a Zeiss LSM 510 laser confocal microscope and analyzed with Carl Zeiss LSM Image software (Jena, Germany). 3D8 scFv-transfected cell lines were assessed by flow cytometry. HeLa cells grown in six-well plates (1×10^5^ cells/well) were pre-incubated in serum-free DMEM for 30 min at 37°C and were either untreated or treated with each inhibitor for 30 min at 37°C before 3D8 scFv (10 mM) treatment. Cells suspended with trypsin were treated once more with 0.1% trypsin for 3 min at 37°C to wash off surface-bound proteins. After washing with ice-cold PBS once, cells were fixed and permeabilized as per the procedures described above. Cells were washed with ice-cold PBS twice, labeled with anti-rabbit 3D8 scFv Ab, followed by TRITC-anti-rabbit Ab, and then analyzed using a FACS Calibur TM (BD Biosciences, San Diego, CA, USA). A total of 1×10^4^ cells were analyzed for each test.

### RNA preparation and quantitative real-time RT-PCR

Quantification of HSV-1 DNA and RNA from cells of SCH07072, muSCH, and HeLa was carried out by harvesting the cells at 2 hours post infection (HPI) with HSV-1, 6.5 HPI, and 25 HPI for immediate early stage, early stage, and late stage, respectively as summarized in Figure 5A. RNA was isolated from cells and mouse tissues (liver, muscle, lung, and brain) using Corezol reagent (CoreBio System) [41]. cDNA was synthesized from 5 µg of total RNA using random hexamers and MMLV reverse transcriptase (SuperBio). All primers were designed using the Primer 3 program. The expression levels of immediate early genes [ICP0 (transcriptional transactivator) and ICP4 (regulatory protein)] [42], [43], early genes [UL9 (replication origin binding) and UL29 (single-strand binding protein)] [44], [45] and late genes [UL19 (capsid protein) and UL38 (capsid assembly)] [46], [47] were analyzed by Rotor-Gene 3000 (Corbett Research, Sydney, Australia) (**Table 2**). The primer efficiency of ICP0, ICP4, UL9, UL29, UL19, and UL38 are 2.081, 1.989, 1.972, 1.916, 1.983, and 2.053, respectively. Each data point represents the average of three individual experiments and the error bars indicate standard errors.

### Immunoblot assays

Virus-infected HeLa cells and SCH07072 cells were lysed using Pro-prep (Intron, Daejeon, Korea). Cell lysates were analyzed by 12% sodium dodecyl sulfate-polyacrylamide gel electrophoresis, transferred to a nitrocellulose membrane using a semi-dry method, incubated with primary antibody (1∶500 dilution for cleaved caspase 3 and INF-beta and a 1∶1000 dilution for PRV gD and HSV) for 12 hr at 4°C, and then incubated with goat anti-mouse or rabbit IgG-HRP conjugate (1∶1000) for 1 hr at 37°C.

### FRET-based DNA and RNA cleavage assays

A 21 base-long ribo-oligonucleotide (500 nM) labeled with 6-carboxyfluorescein (FAM) at the 5′ terminus and a black hole quencher (BHQ) at the 3′ terminus were synthesized (5′ FAM-CGATGAGTGCCATGGATATAC-BHQ 3′), annealed to the non-labeled complementary ribo-oligonucleotide, and then delivered into cells in 96-black-well plates using Lipofectamine transfection reagent (Invitrogen). Immediately after changing the medium, the real-time fluorescence intensity of the cells was read for 2 hr in real time using a fluorescence analyzer at 5 min intervals (Molecular Devices, Sunnyvale, CA, USA).

### Virus yield assay

Vero cells were cultured on 6-well plates (Nunc). Ten-fold serial dilution of the virus samples was prepared in serum-free DMEM media. Each dilution (100 µl) was used to infect Vero cells in duplicate. The virus was allowed to adsorb at 37°C for 1 hr. After 48 hr incubation, the Vero cells were rinsed twice with PBS and stained with crystal violet solution for 5 min. The plates were washed with ddH_2_O and the virus titer was calculated. This plaque analysis included four biological and three technical replicates. Each cell line was infected by virus four times and the numbers of plaques on Vero cells were counted three times, respectively, by using the culture media containing viruses.

### Abzyme test of methylated DNA and chromatin

HeLa genomic methylated DNA and non-methylated DNA were purchased from NEB. Each DNA type (0.2 µg) was treated with 3D8 scFv and DNase I at three different unit concentrations (10×10^−4^, 8.3×10^−4^, and 1.4×10^−4^ U/µl). HeLa chromatin was prepared using the EZ-Zyme Enzymatic Chromatin Prep kit (Upstate Technology, USA) [48]. Prepared chromatin DNA and naked DNA were treated with 3D8 scFv and DNase I (10×10^−4^ U/µl and 8.3×10^−4^ U/µl, respectively). The DNA samples were harvested after 0, 1, 2, and 3 hr of incubation and then each DNA sample was analyzed on an agarose gel for abzyme analysis. The relative quantification of DNA was measured by the height values and performed using GeneSnap 7.09 software (SynGene, UK).

### Neutral red cytotoxicity assay

A neutral red (NR) cytotoxicity assay was performed to test cell viability after treatment with DNase I or 3D8 scFv. DNase I and 3D8 scFv at concentrations ranging from 1.5625×10^−3^ units to 0.05 units were transferred to HeLa cells using a microporator (iNCYTO). Uptake of neutral red dye into intracellular acidic compartments was determined by measuring absorbance at 540 nm.

### Production of 3D8 scFv transgenic (TG) mice

3D8 scFv transgenic mice were produced by Macrogen Co. using standard microinjection procedures. Briefly, fertilized mouse eggs were flushed from the oviducts of superovulated C57BL/6NCrjBgi mice, and male pronuclei were injected with a 2.6 kb fragment (4 ng/µl) of 3D8 scFv that had been obtained by digesting the pcDNA3.1/V5-His B (3D8 scFv) vector with *Nru*I/*Stu*I/*Pvu*I restriction enzymes. The injected eggs were reimplanted in the oviducts of pseudo-pregnant C57BL/6NCrjBgi recipient females. At 3 weeks of age, the animals were tested for the presence of the transgene by PCR analysis of their genomic DNA using forward (5′ CAGAGCTCTCTGGCTAACTAG 3′) and reverse primers (5′ CTGTTGAACAGACTCTGACTG 3′). The 3D8scFv TG founders (F_0_ lines) were mated with wild-type C57BL/6NCrjBgi mice to establish the transgenic lines and produce siblings from the F_0_ lines in a breeding program provided by Macrogen Co. The principles of laboratory animal care (NIH publication 85-23, revised 1986) were followed and all experiments were carried out under the guidelines of the NVRQS, Korea.

### Southern hybridization

Southern blot hybridization was used to confirm integration and determine the copy number of the 3D8 scFv gene in the transgenic mice. Genomic DNA (20 µg) from each transformant was digested with *Eco*RI and *Hind*III restriction enzymes and then the DNA was separated on 1% agarose gels. 3D8 scFv-specific [^32^P]-radiolabeled probes were prepared from the pcDNA3.1V5/HisB-3D8 scFv vector.

### Grouping and viability testing

Three 3D8 scFv TG mouse lines and the wild-type C57BL/6 line were divided into five groups with or without PRV infection (WT-mock, WT-PRV, STG69-PRV, STG90-PRV, and STG135-PRV). Mice were challenged with PRV by intramuscular injection (10 LD_50_). Survival rates in each group were calculated at 12 hr intervals (**Figure S5**). Tissues (muscle and brain) were harvested and stored at −70°C for further RT-PCR and immunohistochemical analyses.

### Immunohistochemical staining

Representative 3 µm-thick brain tissue sections for immunohistochemical analysis were mounted on silane-coated slides as described by Ramos-Vara [49]. Anti-PRV gpD antibody (Jeno Biotech Inc.) was used as the primary antibody (1∶100) and was applied for 60 min at RT. The samples were then treated for color development with the DAB Detection Kit (Ventana Medical Systems, Germany).

### Blood chemistry

Blood collected from STG90 and wild-type mice was centrifuged at 3,000 rpm for 10 min, and plasma was stored at −20°C until analysis. Plasma levels of total albumin, aspartate aminotransferase, alanine transaminase, and other total proteins were analyzed using an automatic blood chemistry analyzer (Selectra II, Merck, Germany).

### Statistical analysis

All analyses were carried out using the GraphPAD Prism program (GraphPAD Software, La Jolla, CA, USA). A one-way analysis of variance and Tukey's post hoc *t*-test were used for statistical analyses. Data are presented as mean ± SE. A p<0.05 was considered significant. The Kaplan-Meier survival analysis was used to compare survival against PRV infection. The statistical *P* value was generated between WT-PRV and STG90-PRV by the log-rank test.

## Supporting Information

Figure S1 **Expression of 3D8 scFv has no effect on cell growth or endogenous gene expression.** **A**. The three cell lines (WT HeLa, SCH07072, and muSCH) showed similar growth curves during a 72 hr culture period. This result indicates that the 3D8 scFv protein is not associated with cell toxicity *in vitro*. **B**. Northern hybridization revealed that two housekeeping genes (GAPDH and actin) and one inducible gene (VEGF) were expressed at the same levels in all three cell lines.
(TIF)

Figure S2 **3D8 scFv has antiviral effects against HSV but DNase I dose not.** **A**. 3D8 scFv and DNase I were transferred to HeLa cells using a microporator (iNCYTO) to investigate cell viability using a neutral red assay. The concentration of each protein was adjusted between 1.5625×10^−3^ and 0.05 units. No cell viability differences were observed between 3D8 scFv and DNase I at concentrations of 3D8 scFv and DNase I up to 0.05 units. **B**. 3D8 scFv and DNase I were detected in both the cytosol and nucleus of HeLa cells under a confocal microscope. Nuclei were stained with DAPI. 3D8 scFv and DNase I were visualized by immunofluorescence using a polyclonal anti-3D8 scFv antibody and monoclonal anti-DNase I antibody, which were visualized with TRITC (Rhodamine). **C**. 3D8 scFv and DNase I were transferred to HeLa cells using a microporator (iNCYTO) followed by HSV infection (MOI 0.1). Measurement of HSV UL19 mRNA levels showed that 3D8 scFv had an 8-fold greater antiviral effect than DNase I. Bars are means ± standard errors.** indicates a significant difference from HeLa cells at *p<0.01* (one-way analysis of variance and Tukey's post hoc *t*-test).
(TIF)

Figure S3 **Molecular characterization of 3D8 scFv-expressing transgenic mice.** **A**. 3D8 scFv transgenic F_0_ mice were identified by genomic polymerase chain reaction (PCR) and ten lines (47, 69, 90, 92, 108, 109, 110, 115, 128, and 135) were selected for further analysis. **B**. Southern blot hybridization showed that lines 69, 90, and 135 had two copies of the 3D8 scFv gene.
(TIF)

Figure S4 **Family tree of 3D8 scFv TG mice and schematic diagram of the PRV challenge protocol.** **A**. The 3D8 scFv TG founders (F_0_ lines) were mated with wild-type C57BL/6NCrjBgi mice to establish transgenic lines and produce siblings from the F_0_ lines. Four F_1_ lines (69 F_1_: 31, 33, 38, and 208) were produced from the F_0_ 69 line. Each of the four 69 F_1_ lines were mated with wild type C57BL/6NCrjBgi mice, resulting in a total of 20 69 F_2_ lines. These lines were used for virus challenge experiments and were named STG69. Three 90 F_1_ lines (90 F_1_: 45, 46, and 162) and five 135 F_1_ lines (135 F_1_: 140, 142, 144, 165, and 166) were produced, and then 17 90 F_2_ lines (STG90) and 24 135 F_2_ lines (STG135) were generated. The F_2_ progeny of the 69, 90, and 135 founder mice were used in the experiments. Males are depicted as circles and females as boxes. B. Each TG line and the WT line were challenged with 10 LD_50_ PRV in the femoral muscle, and the number of live and dead mice was counted every 12 hr for 5 days to investigate survival rates. PRV-infected WT mice exhibited PRV typical disease symptoms beginning 3–5 days post-challenge.
(TIF)

Figure S5 **3D8 scFv has no effect on mouse growth.** No body weight differences were observed between STG90 mice and WT mice (C57BL/6) for 7 weeks after birth (n = 9 mice per genotype and gender). **A**. Weight of female mice. **B**. Weight of male mice. **C**. Semi-quantitative RT-PCR exhibited that one housekeeping gene (GAPDH), two apoptosis genes (K-ras and Bax), and one growth factor gene (VEGF) were expressed at the same levels in wild type and STG90 mice.
(TIF)
