# Correction: A Nucleic-Acid Hydrolyzing Single Chain Antibody Confers Resistance to DNA Virus Infection in HeLa Cells and C57BL/6 Mice

**DOI:** 10.1371/journal.ppat.1004318

**Published:** 2014-07-23

**Authors:** The PLOS Pathogens Staff

In the abstract, the name of cell line appears as SCH7072. The name of this cell line should read as SCH07072.

The Funding statement should be corrected to read as follows: This study was supported, in part, by a grant from the Agenda (No. PJ0102012014) from the Rural Development Administration (RDA) of Korea and the Korea Institute of Ocean Science and Technology project (No. PE99154) of Korea. The funders had no role in study design, data collection and analysis, decision to publish, or preparation of the manuscript.
