# Supplementary figures and images for: A Nucleic-Acid Hydrolyzing Single Chain Antibody Confers Resistance to DNA Virus Infection in HeLa Cells and C57BL/6 Mice
Source: PLoS Pathog. 2014 Jun 26;10(6):e1004208. doi: 10.1371/journal.ppat.1004208 (PMC4072776; doi:10.1371/journal.ppat.1004208)

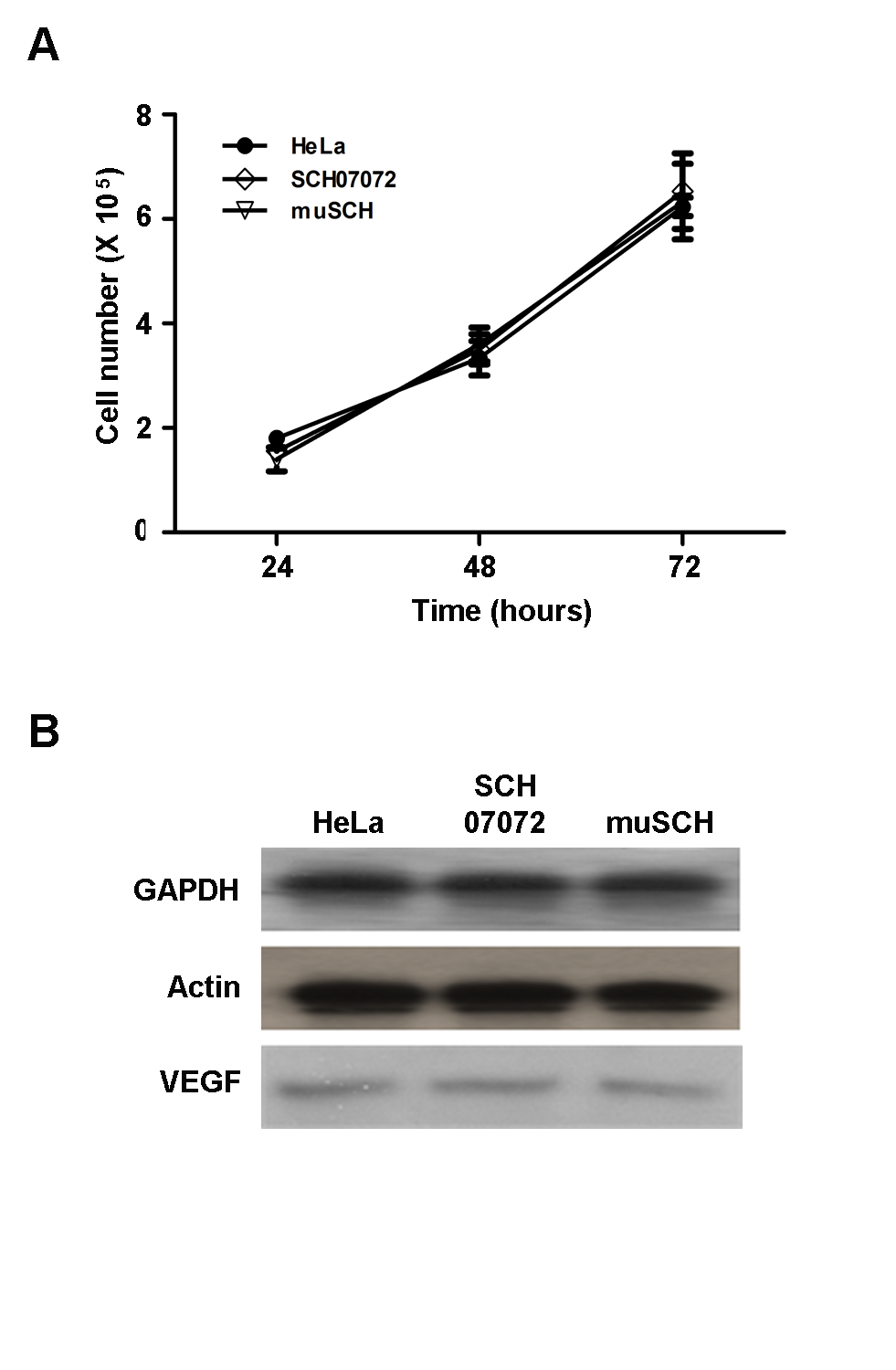

Supplement: Figure S1 — Expression of 3D8 scFv has no effect on cell growth or endogenous gene expression. A. The three cell lines (WT HeLa, SCH07072, and muSCH) showed similar growth curves during a 72 hr culture period. This result indicates that the 3D8 scFv protein is not associated with cell toxicity in vitro. B. Northern hybridization revealed that two housekeeping genes (GAPDH and actin) and one inducible gene (VEGF) were expressed at the same levels in all three cell lines. (TIF) [file ppat.1004208.s001.tif]

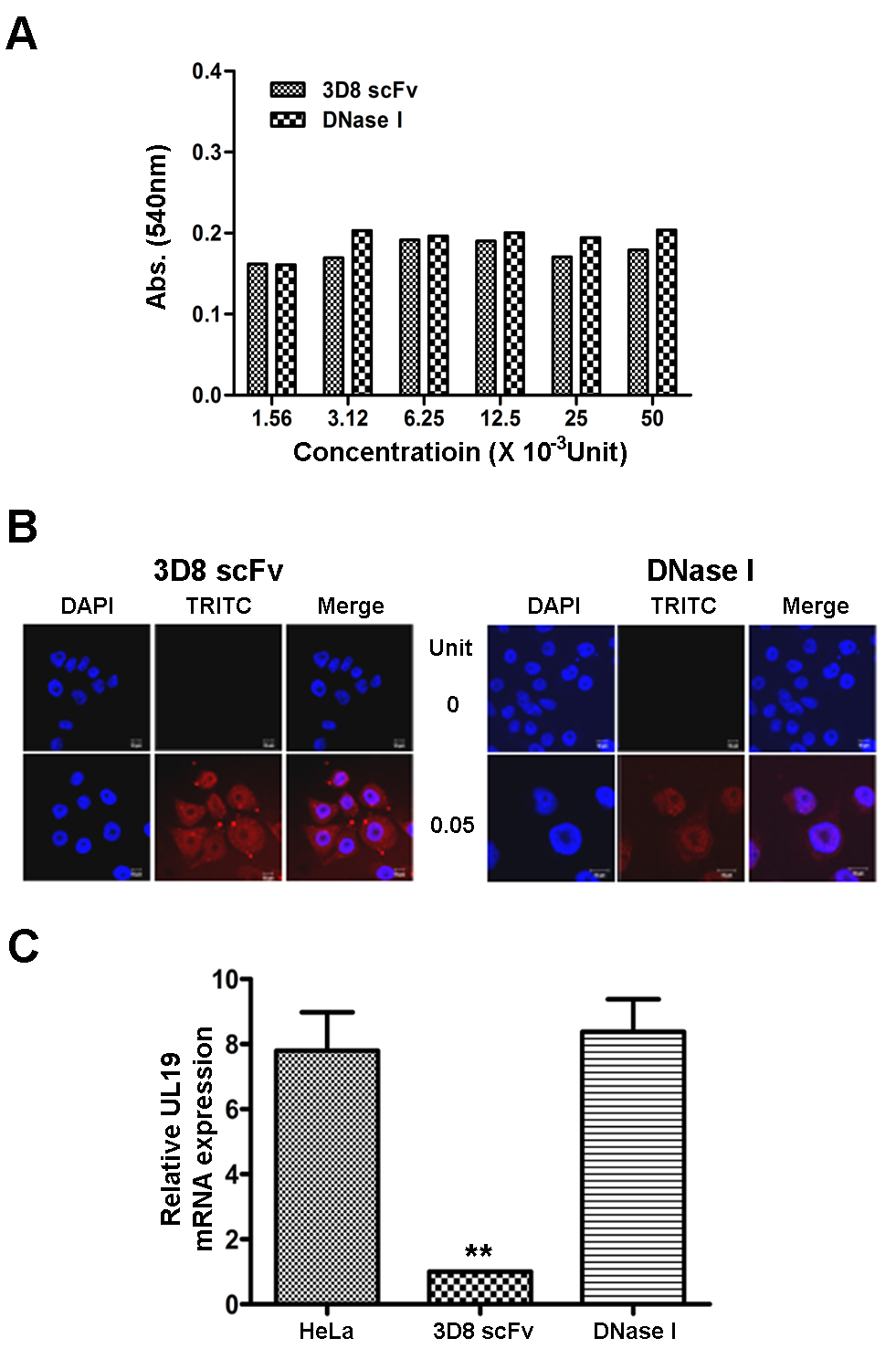

Supplement: Figure S2 — 3D8 scFv has antiviral effects against HSV but DNase I dose not. A. 3D8 scFv and DNase I were transferred to HeLa cells using a microporator (iNCYTO) to investigate cell viability using a neutral red assay. The concentration of each protein was adjusted between 1.5625×10^−3^ and 0.05 units. No cell viability differences were observed between 3D8 scFv and DNase I at concentrations of 3D8 scFv and DNase I up to 0.05 units. B. 3D8 scFv and DNase I were detected in both the cytosol and nucleus of HeLa cells under a confocal microscope. Nuclei were stained with DAPI. 3D8 scFv and DNase I were visualized by immunofluorescence using a polyclonal anti-3D8 scFv antibody and monoclonal anti-DNase I antibody, which were visualized with TRITC (Rhodamine). C. 3D8 scFv and DNase I were transferred to HeLa cells using a microporator (iNCYTO) followed by HSV infection (MOI 0.1). Measurement of HSV UL19 mRNA levels showed that 3D8 scFv had an 8-fold greater antiviral effect than DNase I. Bars are means ± standard errors.** indicates a significant difference from HeLa cells at p<0.01 (one-way analysis of variance and Tukey's post hoc t-test). (TIF) [file ppat.1004208.s002.tif]

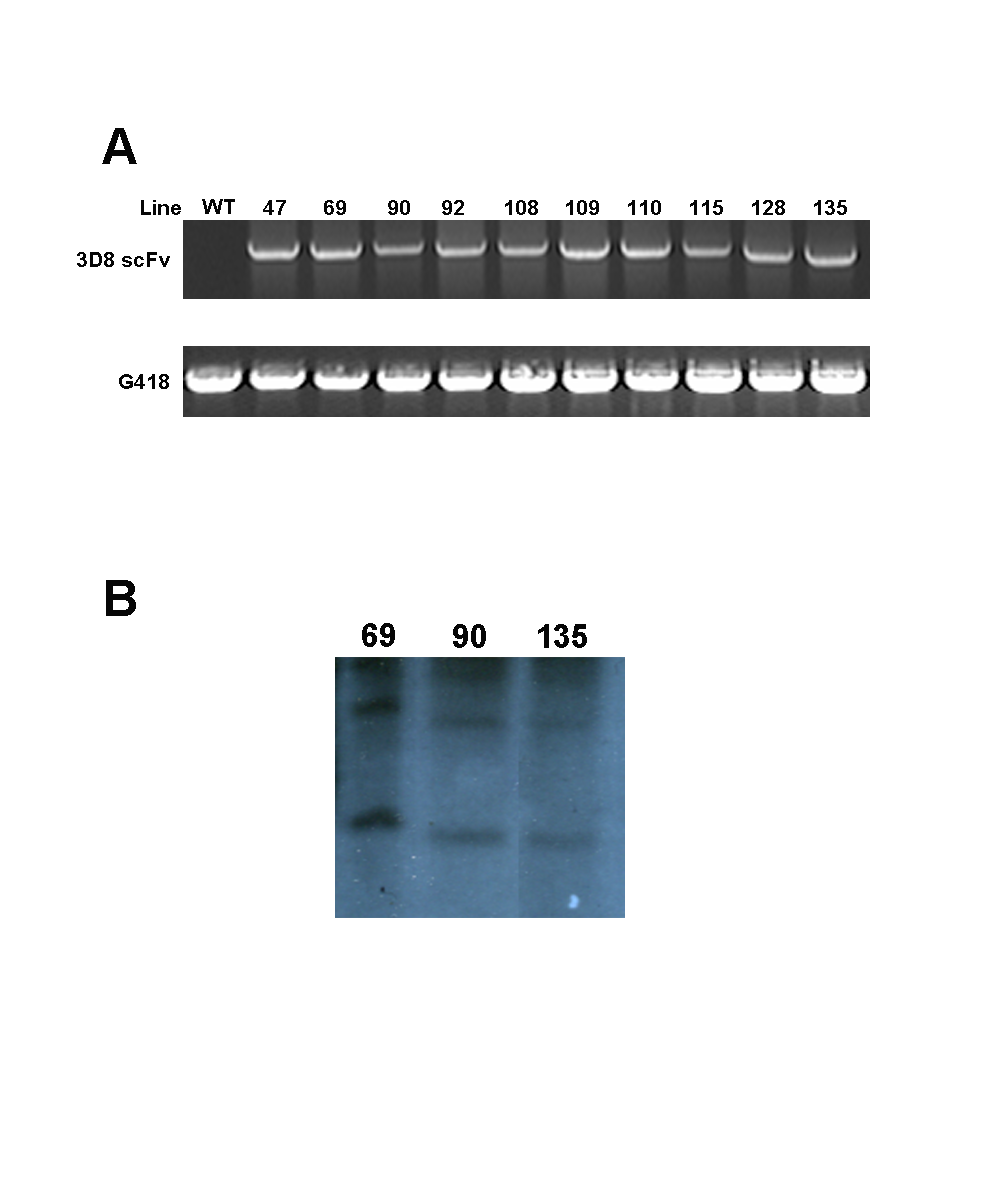

Supplement: Figure S3 — Molecular characterization of 3D8 scFv-expressing transgenic mice. A. 3D8 scFv transgenic F0 mice were identified by genomic polymerase chain reaction (PCR) and ten lines (47, 69, 90, 92, 108, 109, 110, 115, 128, and 135) were selected for further analysis. B. Southern blot hybridization showed that lines 69, 90, and 135 had two copies of the 3D8 scFv gene. (TIF) [file ppat.1004208.s003.tif]

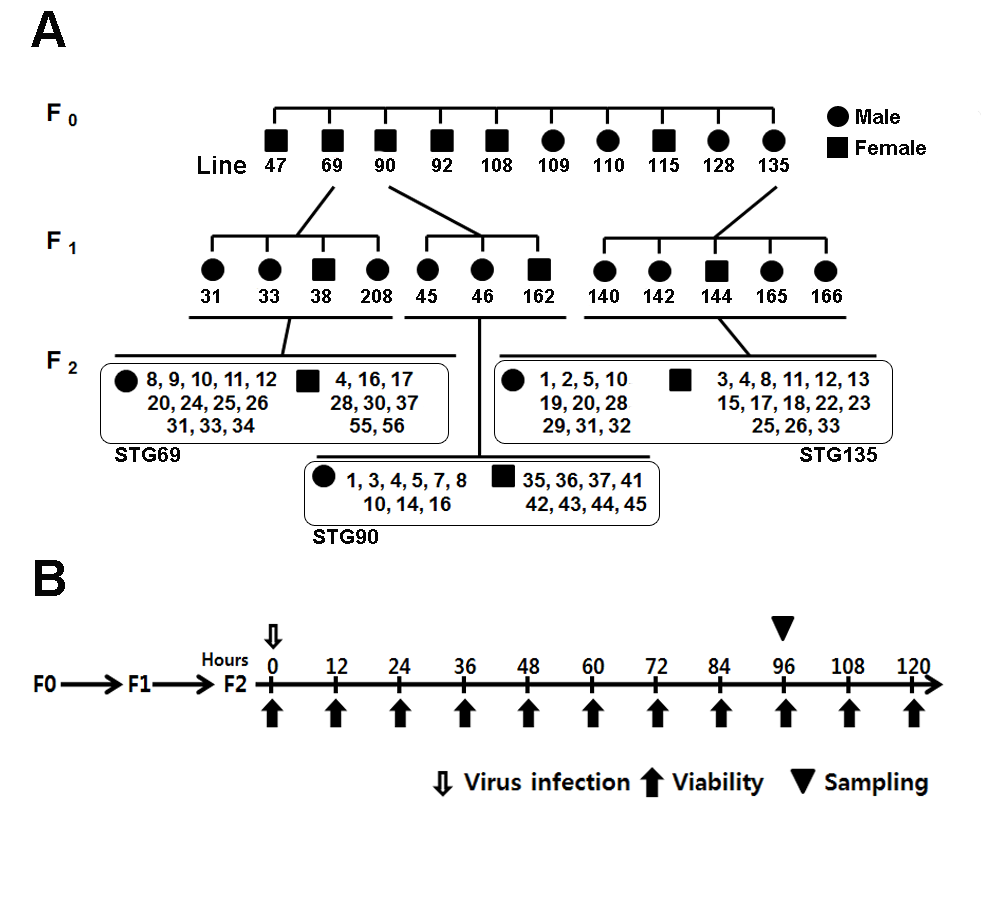

Supplement: Figure S4 — Family tree of 3D8 scFv TG mice and schematic diagram of the PRV challenge protocol. A. The 3D8 scFv TG founders (F0 lines) were mated with wild-type C57BL/6NCrjBgi mice to establish transgenic lines and produce siblings from the F0 lines. Four F1 lines (69 F1: 31, 33, 38, and 208) were produced from the F0 69 line. Each of the four 69 F1 lines were mated with wild type C57BL/6NCrjBgi mice, resulting in a total of 20 69 F2 lines. These lines were used for virus challenge experiments and were named STG69. Three 90 F1 lines (90 F1: 45, 46, and 162) and five 135 F1 lines (135 F1: 140, 142, 144, 165, and 166) were produced, and then 17 90 F2 lines (STG90) and 24 135 F2 lines (STG135) were generated. The F2 progeny of the 69, 90, and 135 founder mice were used in the experiments. Males are depicted as circles and females as boxes. B. Each TG line and the WT line were challenged with 10 LD50 PRV in the femoral muscle, and the number of live and dead mice was counted every 12 hr for 5 days to investigate survival rates. PRV-infected WT mice exhibited PRV typical disease symptoms beginning 3–5 days post-challenge. (TIF) [file ppat.1004208.s004.tif]

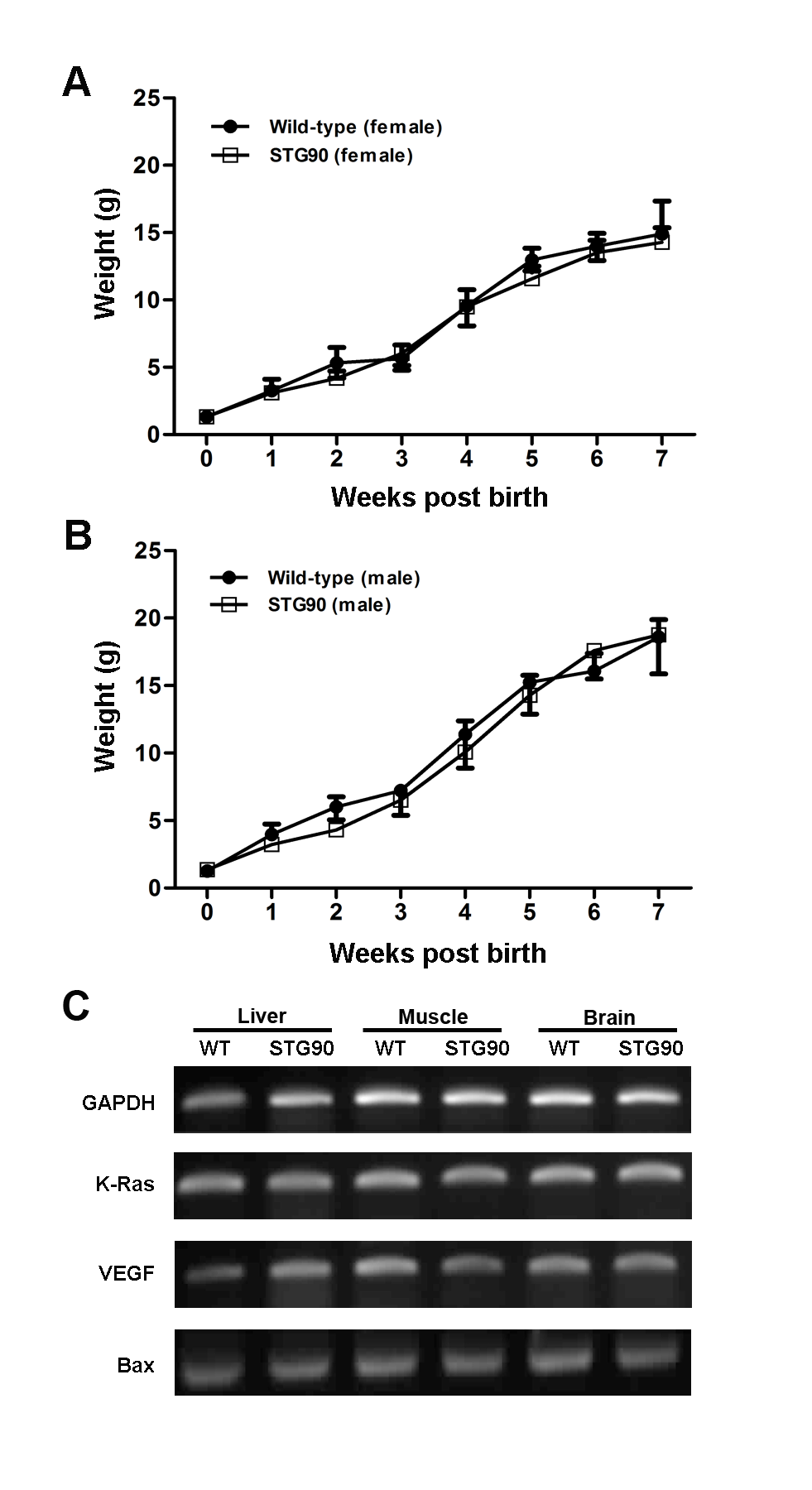

Supplement: Figure S5 — 3D8 scFv has no effect on mouse growth. No body weight differences were observed between STG90 mice and WT mice (C57BL/6) for 7 weeks after birth (n = 9 mice per genotype and gender). A. Weight of female mice. B. Weight of male mice. C. Semi-quantitative RT-PCR exhibited that one housekeeping gene (GAPDH), two apoptosis genes (K-ras and Bax), and one growth factor gene (VEGF) were expressed at the same levels in wild type and STG90 mice. (TIF) [file ppat.1004208.s005.tif]
